# Caregiver lived experiences attempting to follow safe sleep recommendations to sleep their baby in a cot: a qualitative directed content analysis

**DOI:** 10.1017/S1463423626101273

**Published:** 2026-06-04

**Authors:** Carly Grubb, Jeanine Young, Levita D’Souza

**Affiliations:** 1 School of Health, University of the Sunshine Coast, Petrie, Australia; 2 School of Educational Psychology and Inclusion, Monash University, Clayton, Australia

**Keywords:** health disparities, health literacy, infant sleep safety, maternal health, newborn health, public health interventions, sleep health, sudden unexpected death in infancy

## Abstract

**Aim:**

To examine parents’ lived experiences of implementing cot-only advice in a contemporary Australian cohort, assess alignment with the international literature on infant sleep and shared sleep practices, and determine whether explicit risk minimization guidelines address information needs identified by parents.

**Background:**

The risk elimination approach to shared sleeping has dominated public health messaging for decades but has been unsuccessful in reducing Sudden Unexpected Deaths in Infancy (SUDI) rates, while bedsharing rates have remained stable. The risk minimization approach offers an alternative, pragmatic framework for reducing controllable risks.

**Methods:**

A retrospective, secondary, qualitative directed content analysis of free-text responses related to bed-sharing drawn from the 2017 Infant Care Awareness and Routines Evaluation among Queenslanders (I-CARE Qld Study) cross-sectional survey dataset. 3,341 primary caregivers (97% mothers) of infants aged approximately three months, born in Queensland between April and May 2017. For this secondary analysis, free-text responses related to co-sleeping/bedsharing were extracted and analysed qualitatively in line with study objectives.

**Findings:**

Queensland parents expressed a desire for more information and non-judgemental education on improving safety while sharing sleep with their baby. Nearly a third of this cohort reported difficulty following advice to sleep their baby separately citing infant temperament/ needs, breastfeeding and caregiver fatigue or exhaustion as top rationales for this difficulty, aligning with earlier international findings. Some awareness by parents of risk minimization strategies to improve shared sleep safety was evident and in alignment with current Queensland Clinical Guidelines. There is an urgent need for public health messaging on safer infant sleep to better align with the lived experiences of parents and families through the adoption of a universal, proactive risk minimization approach to shared sleep. Co-designing guidance with parents may serve to bridge the gap between idealized and real-world implementation of safer infant sleep strategies.

## Introduction

Across many countries, reductions in Sudden Unexpected Deaths in Infancy (SUDI), including Sudden Infant Death Syndrome (SIDS) and fatal sleep accidents, have slowed and, in some cases, plateaued over the past decade; (Cole *et al*., 2022; Hauck *et al*., 2017; Vincent *et al*., 2023). For example, in Australia rates have remained at approximately 0.3 per 1000 live births since 2014, following earlier declines from 0.6 per 1000 in 2000 (Australian Institute of Health and Welfare, 2026). SUDI is an umbrella term that refers to the sudden and unexpected death of an infant under one year of age, where the cause is not immediately obvious and may be explained after investigation (e.g. accident, illness, or remains unexplained) (Queensland Paediatric Quality Council Prepared by the Infant Mortality Subcommittee, 2025). SIDS is a subset of SUDI and describes those deaths that remain unexplained ever after a thorough investigation, including autopsy, review of the clinical history and examination of the death scene (Krous *et al*., 2004).

Sleep location and environment continue to be an area of focus for efforts to improve infant sleep safety, with evidence showing that the majority of deaths occur in the context of locations and environments with multiple risk factors present (Pease *et al*., 2023). Pillows, soft-bedding (Bombard *et al*., 2018, Moon *et al*., 2022a), falling asleep on a sofa/couch (Blair *et al*., 2009; Rechtman *et al*., 2014, Blair *et al*., 2020, Macfarlane *et al*., 2022), smoke-exposure (Arck Lambert *et al*., 2024; Blair *et al*., 2009; Carpenter *et al*., 2013; Macfarlane *et al.*, 2022; Shipstone *et al*., 2020), bed-sharing with intoxicated caregivers (Arck Lambert *et al*., 2024; Blair *et al*., 2009; Carpenter *et al.*, 2013; Hauck *et al*., 2022; Macfarlane *et al*., 2022), pets or other children (Moon *et al*., 2022a), are common factors identified in child death reviews (Bamber *et al*., 2016; Blair *et al*., 2014; Pease *et al.*, 2023). How best to translate this evidence into safer sleep guidance remains contested, leading to inconsistent and often impractical, public health messaging (Kruse *et al*., 2025). The International Society for the Study and Prevention of Perinatal and Infant Death (ISPID) notes substantial cross-country variation in recommendations (e.g. shared sleeping, pacifier use, sleeping bags) (International Society for the Study and Prevention of Infant Death (IPSID), 2023). A risk elimination approach to shared sleep, led by the American Academy of Pediatrics (AAP), promotes room-sharing without bed-sharing: infants should sleep in the parents’ room, close to the bed, but on a separate infant sleep surface (cot/crib), ideally for the first 6 months, with a clear universal message that parents should never share sleep. (Moon *et al*., 2022a, Grubb *et al*., 2025). Room-sharing on a separate surface reduces SIDS risk by up to 50%, likely by enabling supervision and feeding while avoiding suffocation, strangulation, and entrapment hazards.(Moon *et al*., 2022b).

However, although intended to be protective, its population-level impact has diminished as remaining deaths increasingly involve multiple hazardous sleep factors. Despite dominating public health messaging for several decades, SUDI rates have not fallen further, and have recently increased in the USA in recent years (Centers for Disease Control and Prevention *et al*., 2024). Bedsharing rates remain stable, despite parental awareness of the advice, to always place infants in a separate cot (D’Souza *et al*., 2024; Gilmour *et al*., 2019; Grubb *et al.*, 2025; Sahud *et al*., 2025). Extensive parental accounts show this advice often conflicts with their lived realities, with bedsharing occurring both intentionally and unintentionally for a wide range of reasons (Ball *et al*., 2013; Bombard *et al.*, 2018; Cole *et al.*, 2022; D’Souza *et al.*, 2024; Grubb *et al.*, 2025; Mckenna *et al*., 2017; Pease *et al*., 2025; Sahud *et al*., 2025).

Risk minimization offers an alternative approach, providing practical guidance to reduce controllable risks in shared sleep environments, recognizing that these occur for diverse reasons and that no setting is entirely risk free (Sahud *et al.*, 2025). The Queensland Clinical Guidelines (2022) for safer infant sleep, designed to inform care provision by primary health practitioners, exemplify this risk minimization approach through co-designed guidance that considers both risks and benefits of shared sleep, emphasizing that it is the combination of risk factors which can create hazardous circumstances, rather than the act of shared sleeping being inherently unsafe (Blair *et al*., 2020).

Caring for an infant is a dynamic, complex, and highly variable phenomenon shaped by infant behaviour, parental needs, and social context (D’Souza *et al.*, 2024). In Australia, contemporary infant sleep practices and their alignment with public health safe sleep guidance have been most comprehensively examined through a large population based cross-sectional survey conducted by Cole and colleagues in 2017 (Cole *et al*., 2020a). With responses from 3,341 parents, this study remains the most recent and complete Australian dataset examining infant care practices in relation to safe sleep recommendations.

The uptake of safe sleep messages and prevalence of shared sleep practices (Cole *et al*., 2020a); maternal and infant characteristics and sleep-related factors influencing breastfeeding duration (Cole *et al*., 2020b); parental awareness of safe sleep messages and associated care practices (Cole *et al*., 2021a); and challenges encountered when implementing safe sleep advice (Cole *et al*., 2021b) have been previously reported. Across this body of work, strong and recurring themes were identified, with shared sleep, including co-sleeping and bed-sharing, consistently emerging as a salient issue.

At the time of data collection, one of the six Australian safe sleep recommendations was ‘Sleep baby in their own safe sleeping place in the same room as an adult caregiver for the first six to twelve months’ (Young *et al*., 2012). Although originally intended to support risk minimization across both room-sharing and bedsharing contexts (Young *et al.*, 2012), it was commonly translated to parent education as ‘Sleep baby in a safe cot in the parents’ room’ *(Red Nose Australia, 2024b)* reflecting a risk elimination approach that promotes room-sharing without bed-sharing. This interpretation appears to have arisen from the oversimplified way the guidance was communicated, rather than from the intent of the recommendation itself (Young *et al.*, 2012). While previous analyses have documented behaviours, awareness, and perceived challenges, this dataset enables a deeper exploration of parents’ lived experiences of attempting to follow a recommendation that has been interpreted as being applicable only to cot-based sleep.

The aim of this analysis is to examine parents’ lived experiences of implementing predominantly cot-only advice in a contemporary Australian cohort, assess alignment with findings reported in the international literature on infant sleep and shared sleep practices, and determine whether explicit risk minimization guidelines (e.g. QCG (Queensland Clinical Guidelines *et al*., 2022)) address information needs identified by parents.

## Design

This study was designed as a retrospective, secondary, qualitative directed content analysis of free-text responses related to bed-sharing drawn from the 2017 Infant Care Awareness and Routines Evaluation among Queenslanders (I-CARE Qld Study) (Cole *et al*., 2020a) cross-sectional survey dataset. The qualitative analysis was conducted and reported in accordance with the Consolidated Criteria for Reporting Qualitative Research (COREQ) (Tong *et al*., 2007) to enhance transparency, rigour, and methodological reporting.

## Patient and public involvement

While study participants were not directly involved in the original 2017 study design, the questionnaire was modelled on the 2002 Queensland Infant Care Practice Study (Schluter *et al*., 2002, Cole *et al*., 2020a) with the addition of contemporary questions, and piloted by 30 mothers. This process detailed elsewhere (Cole *et al*., 2020a), facilitated questions that were well defined, clearly understood, and presented in a consistent understandable manner for parents/carers.

### Ethics

Relevant University Human Research Ethics Committee approval was obtained to use de-identified data from the Qld I-CARE study for secondary analysis (S/23/1871).

### Methods: setting and sampling, procedures

The methods for the 2017 Queensland I-CARE study have been reported in detail elsewhere (Cole *et al*., 2020a; 2020b). In brief, the study comprised a cross-sectional survey of primary caregivers of infants aged approximately three months, born in Queensland between April and May 2017. Eligible families (*n* = 10,200) were identified through the Queensland Registry of Births, Deaths, and Marriages, which distributed the survey statewide to home addresses. A total of 3,341 caregivers participated (97% mothers) (See Supplemental File A).

The original dataset was collated and coded in Microsoft Excel (Young, 2017). For this secondary analysis, data relevant to co-sleeping/bedsharing were extracted into separate spreadsheets for qualitative directed content analysis. Inclusion was limited to free-text responses that could address the research questions.

### Data analysis

Directed content analysis addressed four research questions:

1. What additional information about co-sleeping and bedsharing did parents report wanting from healthcare workers?
2. How do parents perceive and navigate recommendations to avoid co-sleeping or bedsharing in the care of their infant?
3. How do the reasons for bedsharing, or inability to avoid it, in a contemporary Queensland cohort compare with those reported in previous international studies?
4. To what extent were co-sleeping-related recommendations identified by parents aligned with current Queensland Clinical Guidelines (QCG) for risk minimization?

Free-text responses were analysed following the process described by Erlingsson and Brysiewicz (2017), to conduct a directed content analysis (Hsieh *et al*., 2005) with meaning units (words or phrases) coded, categorized, and interpreted into themes. A directed approach was taken to validate and/or extend on existing research/theory and validate alignment with current guidance (Hsieh *et al*., 2005). Supplemental Table A details this process. Coding decisions were independently reviewed by a minimum of two team members to enhance trustworthiness and ensure consistency in theme development. Proportions are reported where relevant to indicate relative prominence of themes within this sub-sample, not to estimate population prevalence (Krippendorff *et al*., 2019). Where more than one response per respondent was appropriate to the research question, the denominator is the number of responses.

## Results

### Participant characteristics

Participants comprised a sub-sample of the I-CARE cross-sectional cohort whose responses were identified and coded as co-sleeping related. This content analysis reflects the experiences of predominantly partnered, Australian-born mothers of young, mostly first-born infants, a demographic profile previously described in detail by Cole and colleagues (Cole *et al.*, 2020a). This context is relevant in interpreting how recommendations, particularly those related to infant sleep location, were understood, negotiated, and at times resisted (Cole *et al.*, 2021b).

Sample size varied by question based on the provision of free-text responses to three questions (Table 1, Questions 1.1–1.3).

**Table 1.** Free-text responses to selected I-CARE survey questions (total *n* = 3,341) Table 1 long description.

I-CARE survey question	Any free-text response n (%)	Co-sleeping/ bedsharing- related free-text response n (%)
1.1 ‘*Would you have liked to receive more information about safe sleeping from your healthcare workers (e.g. Doctor, Midwife)? If YES, what would you have liked to receive more information about?”,*	332/3341 (10)	69/332 (21)
1.2 *‘Do you find any of the recommendations difficult to do with baby? If yes, which recommendation(s) do you find difficult and why is it difficult?’*	1199/3341 (36)	356/1199 (30)
1.3 *‘Can you list any key safe sleeping recommendations that reduce the risk of sudden unexpected death in infancy?’ [dot points]*	3117/3341 (93)	164/3117 (5)

### Previously reported sleep practices relevant to present analysis

Previous analyses of the I-CARE dataset demonstrated that shared sleeping was common and frequently unplanned, with substantial discordance between caregivers’ acknowledgement of ‘own sleep space’ recommendations and their own reported practices (Cole *et al.*, 2020a, 2021a). Shared sleeping was also associated with higher breastfeeding continuation, particularly when it occurred routinely (Cole *et al.*, 2020b). These findings provide important context for the present analysis, which examines how caregivers interpret and operationalize sleep-related recommendations in practice.

### Research questions

1. **What additional information about co-sleeping and bedsharing did parents report wanting from healthcare workers?**

In response to the question ‘*Would you have liked to receive more information about safe sleeping from your healthcare workers (e.g. Doctor, Midwife)? If YES, what would you have liked to receive more information about?’* 10% (332/3341) of caregivers said yes, with 21% (69/332) of these responses specifying topics of interest related to co-sleeping/bedsharing. (See Question 1.1, Table 1). Most responses (43/69, 62%) generally reflected a desire for practical guidance on reducing risk during shared sleep. For example, parents requested information on ‘co-sleeping safely’, safe bedsharing’’, and ‘the safest way to co-sleep (Supplemental Table 2). Some parents (*n* = 8/69, 12%) specifically described wanting non-judgemental, realistic discussions that acknowledged the commonality of both intentional and unintentional co-sleeping. Parents emphasized the need for education that moved beyond prohibition to include risk-minimization strategies and open discussion with healthcare providers; illustrative quotes are presented in Table 2.

**Table 2.** Examples of parents’ desire for non-judgemental advice and practical information on co-sleeping/bedsharing Table 2 long description.

Examples of parents’ desire for non-judgemental advice and practical information on co-sleeping/bedsharing
*Understanding that some parents will co sleep so instead of making parents ashamed so they lie about it, educate them on safety measures they can take if they do happen to fall asleep feeding or choose to co sleep* *Explaining how to safely co-sleep & the risks of trying not to co-sleep and accidentally falling asleep in an unsafe manner* *As advised, I tried my best never to fall asleep with my baby in bed, however found it impossible to stay awake during all overnight feeds in the first 4 weeks. Realistic discussion, advice or suggestions around this would‘ve been helpful.* *Safe co-sleeping. Instead of saying do not co-sleep, health workers need to understand that almost all parents do co sleep at some stage and they should be given the knowledge to do so safely.* *Safe ways to co-sleep. I realize this is not considered the best way to sleep and prevent SIDS but sometimes it is necessary and having more information on how to make it as safe as possible would be good.* *More info on co-sleeping how to do it safely most people do it but it’s still considered taboo and if it was more out in the open and talked about people would be better informed* *Would have liked to see more info given on safe co-sleeping, instead of just ‘oh no, you shouldn‘t do that’* *That bedsharing is not taboo or can be done safely.*

Caregivers also sought more detailed information on a range of specific co-sleeping topics, including infant sleep and settling, breastfeeding while co-sleeping, bedding and wrapping, infant positioning, and current research on co-sleeping. Additional information requests addressed reasons co-sleeping can be unsafe, associated risks and benefits, managing reflux, babywearing (i.e. use of infant slings or baby carriers), infant temperament, examples of bedroom set-ups, validation that bedsharing can be safe, guidance for health professionals, and considerations for multiple infants or individual risk factors. Detailed illustrative quotes reflecting these topics are provided in Supplemental File B.

1. **How do parents perceive and navigate recommendations to avoid co-sleeping or bedsharing in the care of their infant?**

**Figure 1.**
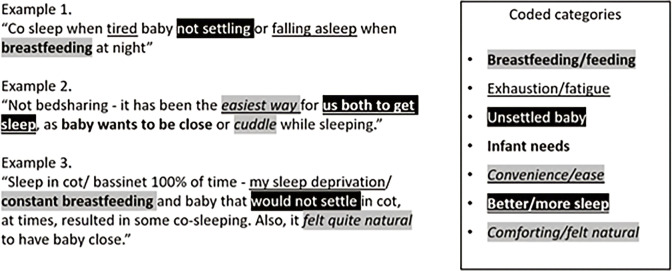
Figure 1 long description. Examples of multi-faceted parent rationales for difficulty in avoiding co-sleeping. Illustrative excerpts from caregiver free-text responses demonstrating overlapping rationales for co-sleeping, including infant needs, breastfeeding/feeding, caregiver fatigue, convenience, and perceptions of comfort or naturalness, with corresponding coded categories shown.

Over a third of respondents (36%, 1199/3341) reported difficulty with at least one safe sleep recommendation in response to the question: ‘*Do you find any of the recommendations difficult to do with baby? If yes, which recommendation(s) do you find difficult and why is it difficult?*’ (Table 1, Question 1.2). Among these responses, nearly one-third (30%, 356/1199) identified challenges related to avoiding co-sleeping or providing a separate infant sleep space. Of those reporting this difficulty, 292 (82%) provided free-text explanations describing their experiences. Many responses were multi-faceted and were therefore coded and categorized and counted in all relevant themes. Examples provided in Figure 1. Negotiating co-sleeping/bedsharing guidance in the context of infant care realities is posed as an overarching/primary theme that captures how parents actively interpret, adapt, or depart from guidance when it conflicts with lived infant caregiving demands.

The most commonly reported reasons for difficulty in avoiding bedsharing were related to infant needs, preferences, or temperament (41%, *n* = 121/292); followed by breastfeeding or feeding considerations (27%, *n* = 80/292); and caregiver exhaustion or fatigue (26%, *n* = 77/292). Other frequently cited factors included achieving better sleep for the infant or caregiver (22%, *n* = 63/292), the perceived comforting nature of co-sleeping for the infant or caregiver (21%, *n* = 62/292); an unsettled or crying baby (20%, *n* = 58/292); and the convenience or ease of co-sleeping (10%, *n* = 30/292, 10%). Illustrative quotations reflecting parent experiences are presented in Table 3. The full extraction table is contained in Supplemental File C.

**Table 3.** Parent rationales for difficulty avoiding bedsharing or sleeping baby separately: illustrative quotes reflecting lived caregiving realities Table 3 long description.

Categories: secondary	Illustrative quotes
1. Infant needs, preferences, or temperament	No co-sleeping [was difficult] because sometimes that’s all baby wants Baby does not sleep in her cot. Separate surface – sometimes baby won’t sleep except if feed laying down together
2. Breastfeeding and feeding generally	Don’t co-sleep: I get sleepy breastfeeding Not cosleeping. When exclusively BF it is better to plan to cosleep than to fall asleep feeding in a chair.
3. Caregiver exhaustion/ fatigue	Exhaustion will lead to having the baby in bed with you Baby sleeping separately – too frequent wake up at night hence I get very exhausted
4. Getting better/more sleep for infant and/or caregiver	Not co-sleeping. Baby sleeps longer and easier to settle if co-sleeping Not sharing the same bed – He sleeps better when he is close to me Sleep on a separate surface – we both sleep well when we co-sleep
4. Co-sleeping was comforting to infant or caregiver	Co sleeping because I like having her next to me during the night. Not bedsharing – it has been the easiest way for us both to get sleep, as baby wants to be close or cuddle while sleeping
5. Unsettled or crying baby	Haven’t always used to cot – practice co-sleeping sometimes if he is very unsettled but follow guidelines for safest way to co-sleep Sleeping baby somewhere that isn’t beside me e.g. bassinet/cot – because he won’t sleep without me beside him. He just cries non-stop and only sleeps all night if co-sleeping
6. Co-sleeping was easier or more convenient	Not to sleep with baby, it is convenient and baby sleeps better Having my child in his own bassinet for all sleep, because when breastfeeding I found it easier lying in bed with my son Not having baby in bed at the beginning. physical injuries from birth prevented easy mobility of mother and it was difficult to get up every 2 hours at night to feed.

1. **How do the reasons for bedsharing, or inability to avoid it, in a contemporary Queensland cohort compare with those reported in previous international studies?**

Table 4 situates findings from the contemporary Queensland I-CARE cohort alongside reasons for bedsharing/co-sleeping identified in two earlier reviews of international literature (Grubb *et al.*, 2025; Salm Ward *et al*., 2015).

**Table 4.** Parent rationales for bedsharing: comparison of queensland cohort with international literature provided Table 4 long description.

Reasons given for bedsharing/ co-sleeping		
Salm Ward, 2015	Grubb *et al*., 2025	Qld I-CARE Dataset
Breastfeeding Comforting- mother or infant Better/More sleep –infant or mother Monitoring Bonding or attachment Environmental reasons	Breastfeeding Comforting for infants/parent Monitoring/safety/protection Better/more sleep (for parent and/or baby) Exhaustion/Fatigue Bonding/attachment/(relationship for both parents)	Infant needs, preferences, or temperament Breastfeeding and feeding generally Caregiver exhaustion or fatigue Better or more sleep for infant and/or caregiver Comforting for infant or caregiver Unsettled or crying baby Easier or more convenient

*Note:* Original themes identified in each review have been retained to ensure accuracy in data reporting and are presented in descending order of most common themes.

There were many similarities between the reasons for bedsharing reported by this contemporary Queensland cohort (predominantly mothers as participants) and those identified in two earlier reviews of the international literature (Grubb *et al.*, 2025; Salm Ward *et al*., 2015). Across all three datasets, breastfeeding, comfort, and achieving better or more sleep were consistently among the most frequently cited reasons. In the Queensland cohort, the most commonly reported reason was infant needs, preferences, or temperament, which was not as evident in the earlier reviews (ranked 8^th^ in Grubb and colleagues’ analysis (Grubb *et al.*, 2025)). Caregiver exhaustion or fatigue was a prominent theme in this cohort, aligning with findings reported by Grubb *et al.* (2025) but not Salm Ward (2015). Other reasons noted in the Queensland data included unsettled or crying babies and the convenience or ease of bedsharing. In contrast, monitoring, protection, and bonding or attachment were reported more prominently in previous reviews (Grubb *et al.*, 2025; Salm Ward *et al*., 2015). Overall, these findings highlight both enduring and context-specific drivers of bedsharing.

1. **To what extent were co-sleeping-related recommendations identified by parents aligned with current Queensland Clinical Guidelines (QCG) for risk minimization?**

Parents were asked *‘Can you list any key safe sleeping recommendations that reduce the risk of sudden unexpected death in infancy?*’ (Table 1, Question 1.3). Of 3,177 respondents (93% of original sample, 3117/3341) to this question, 5% (164/3117) provided responses specifically related to co-sleeping; despite co-sleeping not being explicitly included among the six core recommendations at the time (Cole *et al*., 2020a). More than one response per respondent was possible.

**Figure 2.**
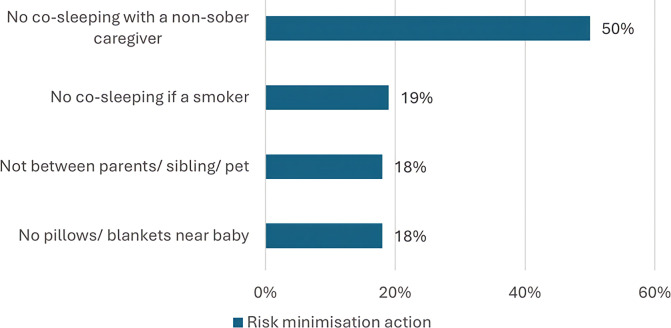
Figure 2 long description. Proportions of respondents reporting the five most common risk minimization practices consistent with Queensland Clinical Guidelines (QCG) for co-sleeping/bedsharing (*n* = 164 free-text responses related to co-sleeping). *Note:* No co-sleeping with non-sober caregiver, *n* = 82/164; No co-sleeping if a smoker, *n* = 32/164; not between parents/sibling/pet, *n* = 30/164; No pillows/blankets near baby, *n* = 30/164.

Approximately half of these co-sleeping responses (*n* = 82, 50%) aligned with Queensland Clinical Guidelines(2022) guidance which recognizes increased risk when a caregiver is impaired by alcohol, medication or drugs. Other parent-identified risks consistent with QCG (Queensland Clinical Guidelines *et al*., 2022) included avoiding infant exposure to smoking (*n* = 32, 19%), preventing shared sleep on couches or sofas (*n* = 17, 10%), positioning the infant to the side of one parent while avoiding placement between parents, and/or sleeping with other children, or pets (*n* = 30, 18%), and minimizing bedding hazards by keeping pillows and adult bedding away from infant or using separate infant bedding or sleeping bags (*n* = 30, 18%). Some parents (*n* = 37, 22%) mentioned ‘practising safe co-sleeping’ without specifying strategies. Figure 2 illustrates the five most common parent responses which are aligned with QCG guidance relating to co-sleeping. The full extraction table is available in Supplemental Table D.

Contraindications to QCG were rare (*n* = 3); but included use of co-sleeping nests, placing babies prone once neck strength is deemed sufficient, or using bedrails; all of which may increase suffocation or entrapment risk. One parent suggested ensuring all adults in the bed are aware of the infant’s presence; not currently mentioned in QCG (Queensland Clinical Guidelines *et al*., 2022) or Red Nose guidance (Cole *et al.*, 2020a, Red Nose Australia, 2024b).

## Discussion

This study aimed to examine parents’ lived experiences of implementing perceived ‘cot-only’ advice in a contemporary Australian cohort, compare how these experiences align with international evidence on infant sleep and shared sleep practices, and explore whether recent guideline risk minimization strategies (e.g. QCG (Queensland Clinical Guidelines *et al*., 2022)) are responsive to information needs identified by parents.

The main themes identified across this directed content analysis (Hsieh *et al*., 2005) were: 1. Certain motivations for bedsharing are universal and reflect fundamental infant- and caregiver needs; 2. Infant needs, preferences, and/or temperament were prominent factors in determining infant care practices related to sleep for this contemporary cohort; 3. Parents need access to non-judgemental, practical support to improve infant sleep safety in shared environments; 4. Current QCG guidelines for safer infant sleep align with parent identified risk minimization strategies for safer shared sleep.

*Certain motivations for bedsharing are universal and reflect fundamental infant-caregiver needs*

By comparing parent-reported rationales across datasets collected in different settings and time periods, this analysis highlights both enduring patterns in why caregivers bedshare and emerging emphases on parents’ lived experiences that may reflect changes in infant care practices, parental expectations, and the interpretation of safe sleep guidance. This comparative approach enables assessment of the extent to which this predominantly Queensland cohort align with, or diverge from, previously synthesized international evidence. Certain motivations for bedsharing are universal and reflect fundamental infant-caregiver needs. Families require advice that is practicable in their lives and circumstances (Barrett *et al*., 2024; Pease *et al*., 2021; Sahud *et al.*, 2025), as infant sleep occurs within a complex interplay of human needs that extend beyond moment-to-moment safety. Breastfeeding and infant feeding, comfort and connection, parent and infant temperament (and other family members), physical environment, relationships, cultural practices, and the practical need for adult sleep across the prolonged infancy period have all been identified as significant, persistent and logical human drivers toward shared sleep, both in this cohort and in the literature spanning the last two decades (Barrett *et al.*, 2024; Crane *et al*., 2016; D’Souza *et al*., 2023; D’Souza *et al.*, 2024; Grubb *et al.*, 2025; Pease *et al*., 2021; Sahud *et al.*, 2025; Salm Ward *et al*., 2014; Salm Ward *et al*., 2015).

These drivers can lead to both deliberate decisions to share sleep, often accompanied by proactive, risk minimization even in the absence of formal guidance (Grubb *et al.*, 2025; Mileva-Seitz *et al*., 2017; Sahud *et al.*, 2025) together with unintentional or spontaneous shared sleep (Hauck *et al*., 2024) which occurs outside of conscious planning and decision-making (Grubb *et al.*, 2025). In the original analysis of this dataset (Cole *et al*., 2020a), 76.9% of infants (*n* = 2520/3341) had shared a sleep surface with another person at some point, and of these, 57.3% of parents reported that it was unplanned. Interestingly, only 356 of these parents subsequently identified the recommendation to sleep their baby in their cot as difficult to follow, suggesting that many may not recognize the relevance of sleep safety guidance for occasional\ or unintentional shared sleep (Pease *et al.*, 2021).

From those who did report difficulty, a clearer picture emerges of the tension between idealized safe sleep recommendations and the realities of infant care, parental sleep needs, and household circumstances. This aligns with themes identified by D’Souza and colleagues (2024) and a recent review by Grubb and colleagues (2025). Infant sleep safety does not occur in a controlled setting and while abstinence-focussed messaging may enhance perceptions of memorability and accessibility (Middlemiss *et al*., 2020; Sahud *et al.*, 2025), it is unlikely to achieve intended outcomes – such as reduced SUDI rates – unless it is acceptable and practical for families (Grubb *et al.*, 2025; Pease *et al.*, 2021; Sahud *et al.*, 2025).

*Infant needs, preferences and/ or temperament were prominent factors in infant care practices related to sleep for this contemporary cohort*

For this cohort, the most prominent motivation for sharing sleep with their infant was the infant’s needs, preferences and/or temperament. This appeared unique to this cohort, as neither of the two earlier reviews identified this motivation as frequently. Together with more frequent reporting of unsettled or crying babies, this finding suggests contemporary caregivers may place greater emphasis on responsive, individualized care in response to infant distress as an immediate driver of shared sleep. This aligns with a recent international review by Liebregts and colleagues (2025) which found that sleep interventions seeking to reduce nighttime parental responding and grounded in Western-centric ideals of solitary, continuous nighttime sleep, may be unacceptable to many families. These findings offer important insight for informing future discussions of infant sleep safety that reflect parents’ lived experiences of settling and sleep with their infant. It is possible that the framing of the survey question – focused on difficulty following cot-only recommendations – elicited different responses than neutral language wording, potentially explaining the lower reporting of ‘bonding’ or ‘attachment’ for this cohort than was evident in the international literature (Grubb *et al*., 2025) as this may not have fit with the respondents rationale for why they slept with their baby in this context or was unrelated to their difficulties following cot-only advice.

Whilst this content analysis did break responses into unit codes in order to quantify the prevalence of reports, as part of the directed content analysis process, looking at the parent responses in their entirety was vital for context as so many overlapped across multiple codes and categories and demonstrated the complex interplay of factors and circumstances culminating in the practice of sharing sleep with their baby. Figure 1 examples provided a dynamic picture of the overall humanness of exploring infant sleep safety when positioned in real world contexts.

*Parents need access to non-judgemental, practical support to improve infant sleep safety in shared environments*

At the time of survey, a risk minimization approach was already evident and recommended in the Australian literature (Young *et al.*, 2012), with the neutrally worded key message, ‘*Sleep baby in their own safe sleeping place in the same room as an adult care-giver for the first six to twelve months’* intended to provide opportunity for conversations regarding both room sharing and bedsharing. Despite this being the wording agreed upon at the 2010 SIDS and Kids (now known as Red Nose Australia) international consensus forum and following extensive review by the SIDS and Kids National Scientific Advisory Group (Young *et al*., 2012), inexplicably, the wording in the parent-facing campaign materials was adjusted and the message oversimplified to: ‘Sleep baby in safe cot in parents’ room’ (Young *et al*., 2012, Cole *et al.*, 2020a). The insertion of the word ‘cot’ shifts the approach back to a risk elimination framing and may close off opportunities to discuss shared sleep safety considerations. Parents in this I-CARE cohort appeared to recognize this and yet many persisted in their requests for more information on the topic. Consistent with previous studies (Pease *et al*., 2021), parents wanted – and expressed a need for – access to non-judgemental conversations, education, and information on safer ways to share sleep (Pease *et al*., 2017). Their requests for more guidance also gave insight into how parents recognize the interwoven nature of sleep safety with the realities and practicalities of infant care with sleep and settling, breastfeeding, positioning, bedding, reflux, risks, and benefits.

*Current QCG guidelines for safer infant sleep align with parent identified risk minimisation strategies for safer shared sleep*

An extremely low number of respondents provided co-sleeping related responses to the request to list any of the key safe sleep recommendations that reduce the risk of SUDI. This is perhaps unsurprising, as the framing of the question may have meant that because most parents knew that the parent-facing guidance supported cot-sleeping, they may have not thought shared sleeping related knowledge or thoughts were relevant and omitted them. Those who did respond showed that their understanding of safe sleep recommendations was very much a list of dos/don’ts (Middlemiss *et al*., 2020; Pease *et al*., 2017) and more heavily favouring the things not to do with more than half of the respondents showing awareness of the increased risk of sharing sleep with an infant if the adult is intoxicated (Arck Lambert *et al*., 2024; Blair *et al*., 2009; Carpenter *et al*., 2013; Hauck *et al*., 2022; Macfarlane *et al.*, 2022; Shipstone *et al.*, 2020). Smaller percentages recognized the increased risk associated with smoking (Blair *et al.*, 2014; Mitchell *et al*., 2017, Blair *et al.*, 2020, Arck Lambert *et al*., 2024; Hauck *et al*., 2022; Macfarlane *et al.*, 2022), and sharing sleep on a couch/ sofa (Blair *et al*., 2009; Blair *et al*., 2014; Macfarlane *et al*., 2022; Rechtman *et al.*, 2014), as well as practical actions to minimize risk, such as the need to keep bedding and pillows away from baby (Bombard *et al*., 2018, Moon *et al*., 2022b) and to not sleep baby between people (Tappin *et al*., 2005), or next to siblings or a pet (Moon *et al.*, 2022a). All of these top 5 responses align with the Queensland Clinical Guidelines for Safer Infant Sleep (Queensland Clinical Guidelines *et al*., 2022). While these guidelines did not exist at the time of the survey, they do provide a useful evidence-based, benchmarking framework to examine the practical steps parents take in an attempt to improve safety in the shared sleep environment. Vitally, this data also provides a checkpoint to see if the guidelines have adequately responded to parent identified needs. One response highlighted explicit advice that could be considered in future Queensland Clinical Guideline iterations: ensuring all adults in the bed are aware of the infant’s presence. This recommendation is consistent with guidance from the Australian College of Midwives (ACM) (Australian College of Midwives *et al*., 2014) and Australian Breastfeeding Association (ABA) (Australian Breastfeeding Association *et al*., 2022) and is supported by evidence from child death reviews (Queensland Paediatric Quality Council Prepared by the Infant Mortality Subcommittee, 2025), although it is not currently included in formal Red Nose guidance (Red Nose Australia, 2024a).

Families need to know they are likely to fall asleep with their baby whether they intend to or not (Grubb *et al.*, 2025; Hauck *et al*., 2024; Pease *et al*., 2021; Pease *et al.*, 2023; Sahud *et al.*, 2025). They also need to know how to ‘*prepare to share’* to provide practicable actions to minimize controllable risks ahead of time (Grubb *et al.*, 2025). This can be facilitated by health professionals involved in primary care and health promotion taking a universal, proactive, neutrally worded risk minimization approach to safer shared sleep, integrated at the core of broader safer infant sleep guidance. The Queensland Clinical Guidelines (2022) and University of Bristol’s Baby Sleep Project (Pease *et al*., 2025; University of Bristol *et al*., 2025) provide evidence-based models to guide this.

Findings from this study confirm that this localized, contemporary Australian cohort have echoed the expressed need for a risk minimization approach to safer shared sleep shared by their international peer voices in the existing literature on the topic. To move this forward, research efforts need to focus on working with parents as experts in their own lives and co-designing public health messages, resources, and campaigns that are fit-for-real-life-purposes. Attention should also be paid to the monitoring and evaluation of the effectiveness of these approaches in reducing SUDI rates over-time, with continual refinement and iteration to ensure messages and tools are appropriate and accessible to the families they serve.

### Strengths and limitations

This retrospective secondary analysis draws on a large cross-sectional cohort dataset providing rich, detailed, and localized insights that enable findings from the international literature to be contextualized within the Queensland setting. The use of the Queensland Registry of Births, Deaths, and Marriages, which purports to capture over 98% of all Queensland births, strengthened the sampling framework and ensured broad population coverage, supporting representativeness of the birthing population at that time. In addition, use of an existing dataset was both time- and cost-effective and reduced ethical and logistical complexities associated with primary data collection, minimizing participant burden.

The directed content analysis methodology provides a robust and systematic interpretation of the content of text data through coding and the identification of patterns and themes (Hsieh *et al*., 2005). Limitations associated with the directed approach to content analysis were evident as there were potentially underexplored opportunities for different/deeper connections between categories and themes that were not the focus of this analysis. To enable comparisons to be drawn across this cohort and the international literature and cross-check alignment with the Queensland Clinical Guidelines, intentionally pre-determined focuses for coding may have restricted the identification of alternative options for coding and therefore categories and themes.

Several other limitations should be acknowledged. Although this remains the most recent largest Australian dataset on infant care practices and safe sleep, the data are now more than eight years old. While it is uncertain whether similar findings would be observed if repeated today, strong consistency with the most recent literature (Grubb *et al*., 2025) supports ongoing relevance of the findings. Shared sleep practices were captured as a sub-component of a broader survey rather than as a primary focus, meaning some variables may lack depth and data collected for other purposes may introduce inaccuracies and inconsistencies. This may be further amplified by the omission of explicit references to shared sleeping in parent-facing recommendations at the time of data collection, potentially influencing participant reporting. Whilst the term ‘parent’ is used throughout this study, 97% identified as mothers, limiting the applicability of and as such, findings to fathers or other caregivers.

## Conclusion

The strong concordance between this analysis and the contemporary international literature reinforces the robustness of the results and strengthens the case for universal, risk-minimization approaches to shared sleep safety, both locally and more broadly, given the consistency of community need. These findings underscore the urgent need for public health messaging on safer infant sleep to better align with the lived experiences of parents and families through the adoption of a universal, proactive risk minimization approach to shared sleep. The QCG guidelines provide an evidence-based framework that is responsive to the practical realities of infant care. By co-designing guidance that meaningfully engages parental expertise and lived experiences, policy-makers, researchers, and clinicians can help to bridge the gap between idealized and real-world practice, supporting safer sleep for infants wherever and whenever it occurs.

## Supporting information

10.1017/S1463423626101273.sm001 Grubb et al. supplementary material Grubb et al. supplementary material

## Data Availability

The datasets used and/or analysed during the current study are not available publicly as this was not specified in the original ethical approval request; however, they may be available on reasonable request. Requests to access datasets should be directed to: JY.

## Supplementary material

The supplementary material for this article can be found at https://doi.org/10.1017/S1463423626101273.

## Table 1. Long description

The table presents data on free-text responses to three selected I-CARE survey questions related to safe sleeping recommendations and co-sleeping. It includes three rows and four columns. The columns are labeled ‘I-CARE survey question’, ‘Any free-text response n (%)’, and ‘Co-sleeping/bedsharing-related free-text response n (%)’. The rows detail specific survey questions and the corresponding response data. Question 1.1 asks if respondents would have liked more information about safe sleeping from healthcare workers, with 10 percent of respondents indicating they would like more information and 21 percent of those mentioning co-sleeping. Question 1.2 asks about the difficulty of recommendations, with 36 percent finding them difficult and 30 percent of those mentioning co-sleeping. Question 1.3 asks for key safe sleeping recommendations to reduce the risk of sudden unexpected death in infancy, with 93 percent providing responses and 5 percent mentioning co-sleeping.

Navigate back to Table 1..

## Table 2. Long description

A table with four rows and three columns presents data on parents’ desire for non-judgmental advice and practical information on co-sleeping and bedsharing. The columns are labeled ‘Did’, ‘N 1’, and ‘Bucket’. The rows provide specific data points: Row 1: Did, 9; N 1, 28.221196; Bucket, bucket underscore 2. Row 2: Did, 1; N 1, 28.221196; Bucket, bucket underscore 3. Row 3: Did, 1; N 1, 28.221196; Bucket, bucket underscore 4. Row 4: Did, 1; N 1, 28.221196; Bucket, bucket underscore 5. The table highlights the percentage of caregivers who desired more information on safe sleeping and the specific topics of interest related to co-sleeping and bedsharing.

Navigate back to Table 2..

## Figure 1. Long description

The table presents examples of multi-faceted parent rationales for difficulty in avoiding co-sleeping. It includes illustrative excerpts from caregiver free-text responses demonstrating overlapping rationales for co-sleeping, such as infant needs, breastfeeding or feeding, caregiver fatigue, convenience, and perceptions of comfort or naturalness. The table has two main sections: examples and coded categories. The examples section contains three rows, each with a unique excerpt highlighting different reasons for co-sleeping. The coded categories section lists the categories corresponding to the excerpts, including breastfeeding or feeding, exhaustion or fatigue, unsettled baby, infant needs, convenience or ease, better or more sleep, and comforting or felt natural. Each excerpt is highlighted with specific phrases that align with the coded categories, showing how different factors intersect in caregivers’ decisions.

Navigate back to Figure 1..

## Table 3. Long description

A table with six rows and two columns. The first column lists categories of reasons for difficulty avoiding bedsharing, including infant needs, breastfeeding, caregiver exhaustion, better sleep, comforting nature, unsettled baby, and convenience. The second column provides illustrative quotes from parents reflecting their lived caregiving realities. The table highlights various challenges and considerations parents face, such as infant preferences, feeding practices, and personal fatigue, which influence their decisions regarding bedsharing.

Navigate back to Table 3..

## Table 4. Long description

The table compares reasons for bedsharing or co-sleeping across three studies: Salm Ward, 2015; Grubb et al., 2025; and the Qld I-CARE Dataset. The table has three columns and four rows of data. The columns are labeled with the study names. The rows list reasons such as breastfeeding, comforting, monitoring, bonding, and environmental reasons. Each study provides specific rationales for bedsharing, highlighting common themes like better sleep, comfort, and bonding. The Qld I-CARE Dataset includes additional reasons like caregiver exhaustion and unsettled or crying babies.

Navigate back to Table 4..

## Figure 2. Long description

The bar graph compares the proportions of respondents reporting the five most common risk minimization practices consistent with Queensland Clinical Guidelines for co-sleeping. The graph features five horizontal bars, each representing a different risk minimization action. The x-axis ranges from 0 to 60 percentage, while the y-axis lists the risk minimization actions: No co-sleeping with a non-sober caregiver, No co-sleeping if a smoker, Not between parents/sibling/pet, and No pillows/blankets near baby. The bar for No co-sleeping with a non-sober caregiver is the longest, reaching 50 percentage. The bars for No co-sleeping if a smoker, Not between parents/sibling/pet, and No pillows/blankets near baby are shorter, each reaching 19 percentage, 18 percentage, and 18 percentage respectively. The color scheme uses a single dark blue color for all bars. The data represents responses from 164 free-text responses related to co-sleeping. All values are approximated.

Navigate back to Figure 2..
