# Supplementary material for: Caregiver lived experiences attempting to follow safe sleep recommendations to sleep their baby in a cot: a qualitative directed content analysis
Source: Prim Health Care Res Dev. 2026 Jun 4;27:e61. doi: 10.1017/S1463423626101273 (PMC13247794; doi:10.1017/S1463423626101273)
Supplement: Grubb et al. supplementary material [file S1463423626101273sup001.docx]

Supplemental File A: 2017 Survey tool


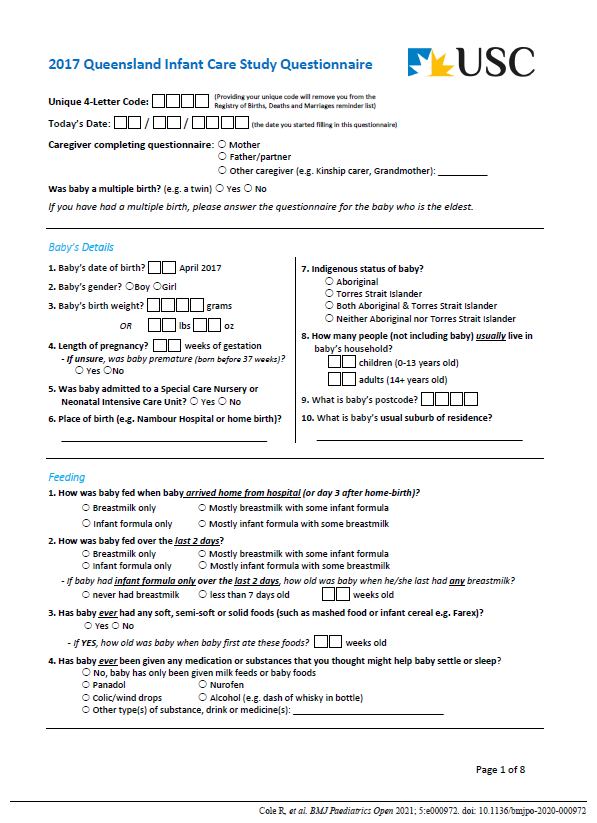


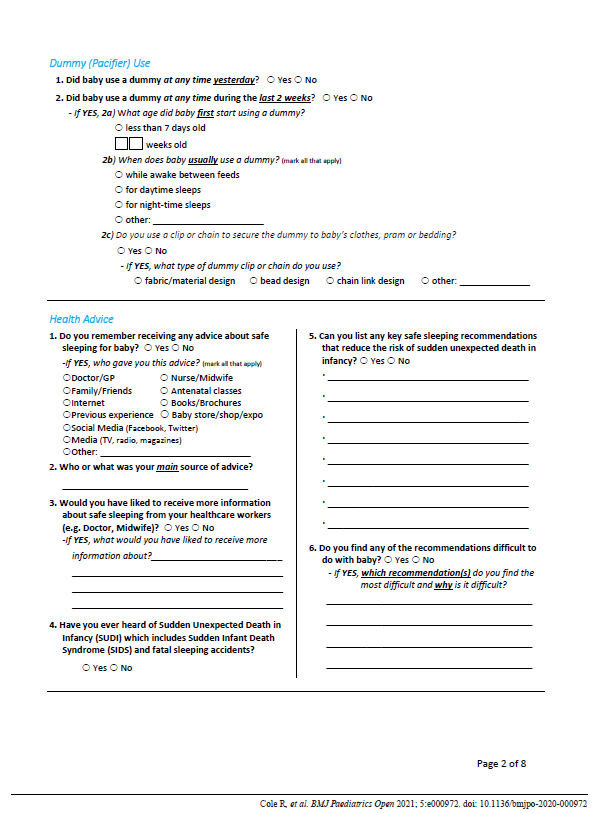


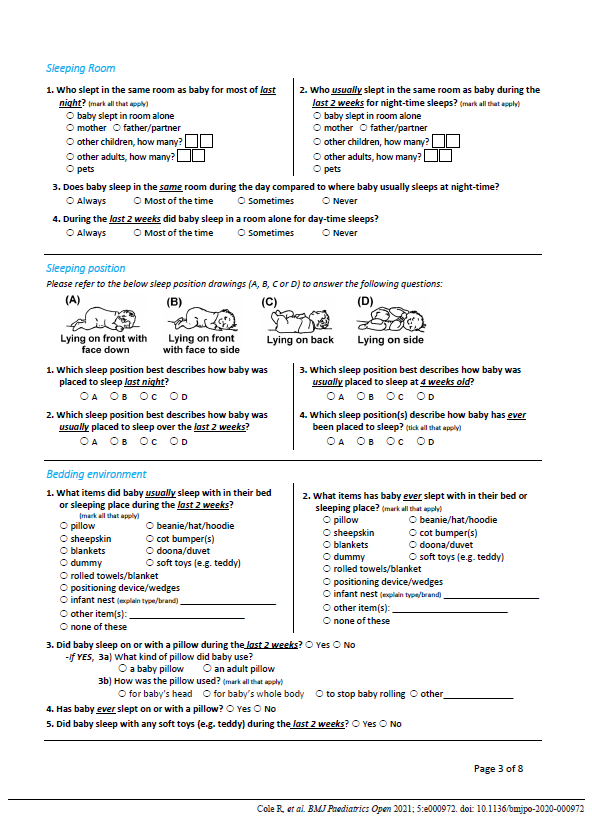


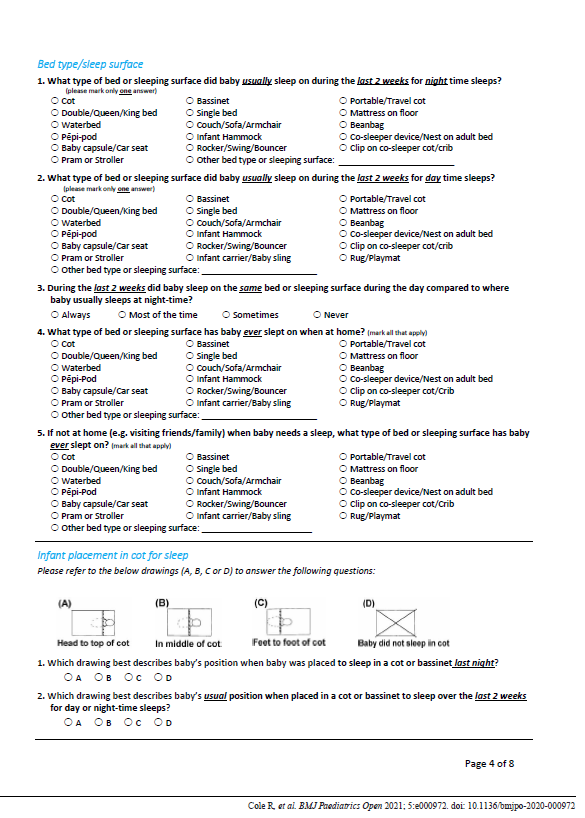


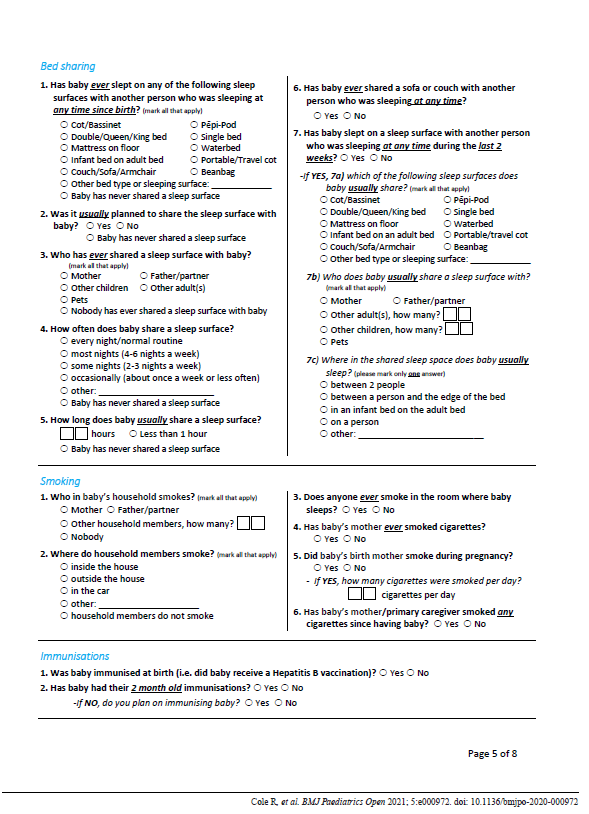


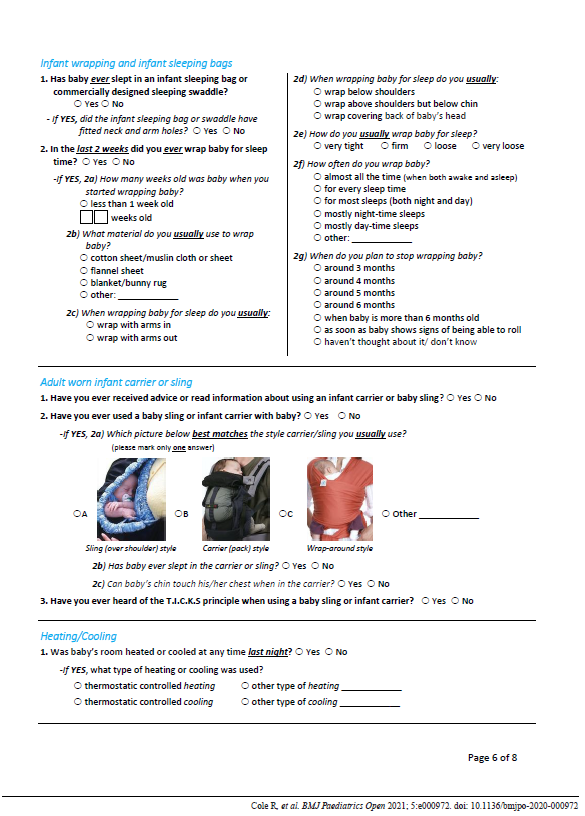


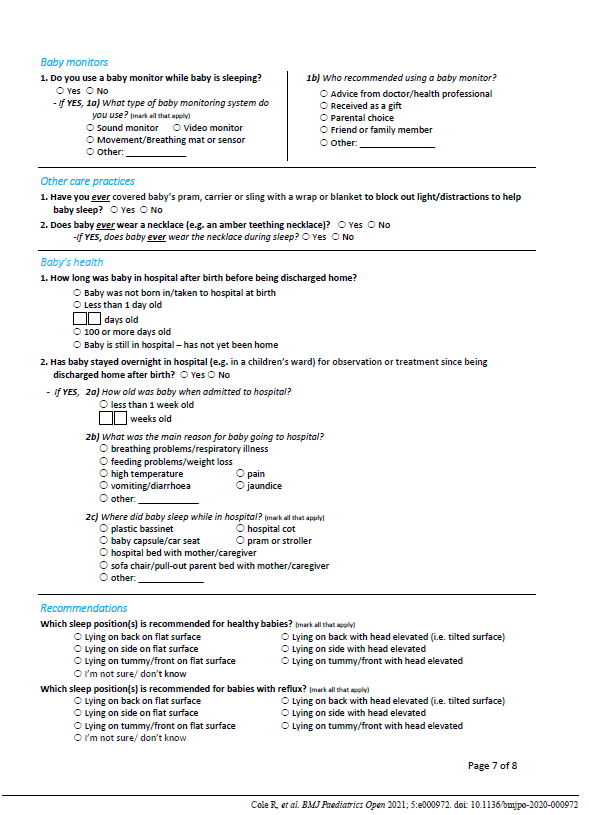


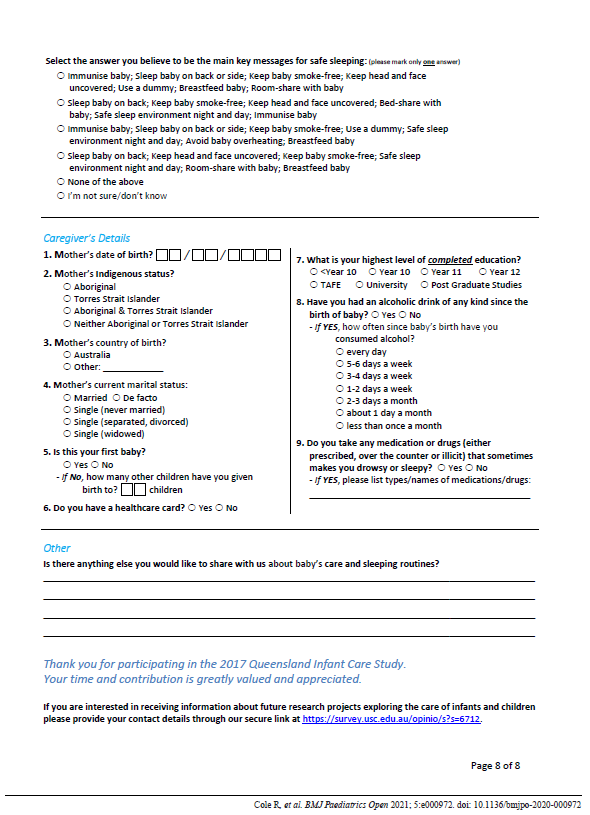


Supplemental Table A: Directed content analysis abstraction process flowchart

| Abstraction of data during content analysis following the process from Erlingsson and Brysiewicz ^38^ | **Theme** | Desire for access to more non-judgemental advice and practical information about shared sleep | Agreement with international literature with additional emphasis on meeting infant needs and temperament | Common alignment with QCG guidance, very few exceptions. Evidence of ambiguity in what ‘safer co-sleeping’ means to parents. |
| --- | --- | --- | --- | --- |
|  | **Category**  ***Building on existing research / theory^39^** | General risk minimisation  Education/ conversation  Specific situations/ scenarios | Categorised into groups from earlier analysis of reasons for bedsharing. Additional categories added as needed. | Categorised as in line, not in line or ambiguous with the QCG guidelines and risk minimisation guidance |
|  | **Code** | General asks- more specific asks | Coded using key words from earlier analysis of reasons for bedsharing. Additional codes added as needed. | Coded looking for key risk and/ or risk minimisation elements |
|  | **Condensed meaning unit** | Free text responses related to co-sleeping/ bedsharing | Free text responses related to co-sleeping/ bedsharing | Free text responses related to co-sleeping/ bedsharing |
|  | **Meaning unit** | Entire free text responses to the question | Entire free text responses to the question | Entire free text responses to the question |
| **Research Question(s)** | | RQ 1.What additional information about co-sleeping and bedsharing did parents report wanting from healthcare workers? | RQ 2.How do parents perceive and navigate recommendations to avoid co-sleeping or bedsharing in the care of their infant? | RQ 4.To what extent were co-sleeping-related recommendations identified by parents aligned with current Queensland Clinical Guidelines (QCG) for risk minimisation? |
|  |  |  | RQ 3.How do the reasons for bedsharing, or inability to avoid it, in a contemporary Queensland cohort compare with those reported in previous international studies? |  |
| **I-CARE survey Question** | | 1.1 ‘*Would you have liked to receive more information about safe sleeping from your healthcare workers (e.g. Doctor, Midwife)? If YES, what would you have liked to receive more information about?”,* | 1.2 *‘Do you find any of the recommendations difficult to do with baby? If yes, which recommendation(s) do you find difficult and why is it difficult?’* | 1.3 *‘Can you list any key safe sleeping recommendations that reduce the risk of sudden unexpected death in infancy?’ [dot points]* |

Supplemental Table B:

Table 6 RQ1: What specific information about co-sleeping/ bedsharing did parents request?

| Kind of info theme | Free-text |
| --- | --- |
| General request information for co-sleeping/ bed sharing safety  N=43/69  62% | 1. Co-sleeping safely 2. Safe bed sharing 3. Co-sleeping 4. Co-sleeping safely 5. Safe co-Sleeping methods 6. Co-sleeping 7. Co-sleeping 8. Safe ways to co-sleep. I realise this is not considered the best way to sleep and prevent SIDS but sometimes it is necessary and having more information on how to make it as safe as possible would be good. 9. Safe co-sleeping 10. Safe co-sleeping and bedsharing information 11. Pros and Cons of Co-sleeping. Safe ways to Co-sleep 12. Co-sleeping safety 13. How to safe co-sleep 14. How to co-sleep safely 15. how to sleep baby safe tips for co-sleeping 16. Safe bed sharing 17. Co-sleeping, baby wearing 18. More information about safe methods of co-sleeping and bed sharing 19. safe bed sharing 20. Co-sleeping 21. Co-sleeping 22. Cosleeping safely 23. Safe cosleeping 24. Some More training to take home about safe sleeping. I was a bit paranoid that my baby still sleeps next to me. 25. Safe co-sleeping 26. Safe bed sharing 27. Co-sleeping safely 28. Safest way to co-sleep (along with the SIDS recommendations) 29. would have liked to see more info given on safe co-sleeping, instead of just 'oh no, you shouldn't do that' 30. co-sleeping 31. How co-sleeping can be made as safe as possible 32. Information Re: Co-sleeping 33. safe ways to co-sleep 34. safe bed sharing 35. Co-sleeping 36. Safe sleeping for co-sleeping 37. safe co-sleeping 38. how to co-sleep safely, I was so scared about it at first, but it saved us 39. co-sleeping/bedsharing 40. co-sleeping 41. Safe sleeping sharing bed 42. co-sleeping 43. maybe more about 'safe' co-sleeping |
| Information/ Education/ Conversation  Non- judgmental, conversation and education about safer co-sleeping, risk minimisation for both intentional and unintentional shared sleep  N= 8/69  12% | 1. Understanding that some parents will co sleep so instead of making parents ashamed so they lie about it, educate them on safety measures they can take if they do happen to fall asleep feeding or choose to co sleep 2. explaining how to safely co-sleep & the risks of trying not to co-sleep and accidentally falling asleep in an unsafe manner 3. As advised, I tried my best never to fall asleep with my baby in bed, however found it impossible to stay awake during all overnight feeds in the first 4 weeks. Realistic discussion, advise or suggestions around this would've been helpful. 4. Safe co-sleeping. Instead of saying do not co-sleep, health workers need to understand that almost all parents do co sleep at some stage and they should be given the knowledge to do so safely. 5. Safe ways to co-sleep. I realise this is not considered the best way to sleep and prevent SIDS but sometimes it is necessary and having more information on how to make it as safe as possible would be good. 6. more info on co-sleeping how to do it safely most people do it but its still considered taboo and if it was more out in the open and talked about people would be better informed 7. would have liked to see more info given on safe co-sleeping, instead of just 'oh no, you shouldn't do that' 8. That bedsharing is not taboo or can be done safely. |
| Specifics  Infant sleep/settling, breastfeeding and co-sleeping  N= 3/69  4% | 1. Sleeping, breastfeeding, Co sleeping 2. Normal expectations around sleep, including lengths and times and how co-sleeping might be safely implemented. 3. Room temperature, settling and reflux, safe co-sleeping. |
| Specifics  Co-sleeping, bedding and wrapping  N= 3/69  4% | 1. Co-sleeping, bedding, swaddling - Risks and safe practices of these 2. swaddling and co-sleeping - it is not recommended to co-sleep but the reality is most parents at some stage do. 3. Appropriate bedding, co-sleeping risks/benefits |
| Specifics  Co-sleeping and positioning  N= 3/69  4% | 1. Co-sleeping and side sleeping safely 2. safe sleeping when baby barely sleeps/only sleeps upright/only sleeps with caregivers 3. if baby will not settle on their back and will only sleep when held, come info about co-sleeping would've been appreciated |
| Specifics  Co-sleeping research  N= 3/69  4% | 1. More accurate info re safe co-sleeping as per Bed Sharing review by Blair et al. 2014. 2. Co-sleeping studies (i.e. SIDS non-existent in Eastern parts of world) 3. most recent research and changes in advice - eg our paediatrician says co-sleeping now ok. I wish I'd been told that for my first baby. |
| Information/ Education/ Conversation  Reason co-sleeping can be unsafe  N= 2/69  2% | 1. Why it's unsafe to co-sleep 2. Safe sleep guidelines: I do not cosleep however I found the lack of information as to why you shouldn't was worrying. |
| Information/ Education/ Conversation  Risks/ benefits of co-sleeping  N= 2/69  2% | 1. Pros and Cons of Co-sleeping. Safe ways to Co-sleep 2. Appropriate bedding, co-sleeping risks/benefits |
| Specifics  Reflux and co-sleeping  N= 2/69  2% | 1. Reflux and sleeping How to tuck a baby into a large cot Co-sleeping 2. Room temperature, settling and reflux, safe co-sleeping. |
| Specifics  Co-sleeping and babywearing  N= 1/69  1% | 1. Co-sleeping, baby wearing |
| Specifics  Co-sleeping and infant temperament  N= 1/69  1% | 1. safe sleeping when baby barely sleeps/only sleeps upright/only sleeps with caregivers |
| Specifics  Individual examples of bedroom set-ups for co-sleeping  N= 1/69  1% | 1. specific examples/feedback on own bedroom set up for co-sleeping |
| Information/ Education/ Conversation  Validation that bedsharing is safe  N= 1/69  1% | 1. Just to confirm and validate that bed-sharing is natural and safe |
| Information/ Education/ Conversation  Improved education for Health Professionals  N= 1/69  1% | 1. More information safe bedsharing guidelines. Nurse and doctors need to be educated on the benefits |
| Information/ Education/ Conversation  The impact of the presence or absence of individual risk factors and SIDS and co-sleeping  N= 1/69  1% | 1. I would like more research done in regard to how different individual factors lead to SIDS not just having them all lumped together. For example, how likely SIDS in relation to co sleeping only (if you don't drink alcohol or smoke or are obese etc). |
| Information/ Education/ Conversation  Midwives not pushing co-sleeping  N= 1/69  1% | 1. My midwives kept pushing co sleeping on me which I didn't agree with |
| Specifics  Co-sleeping and twin sleep safely  N= 1/69  1% | 1. Co-sleeping and twin sleeping safety |

Supplemental Table C: Data extraction table used for content analysis of the free-text responses for RQ 1.2: parent rationales for difficulty avoiding bedsharing or sleeping baby separately

| ***Reason for bedsharing theme*** | ***ICP Data-Reasons why recommendation to not co-sleep/ bedshare difficult to follow***  ***N =356 respondents – 64 who gave no reasons = 292 respondents*** |
| --- | --- |
| Infant needs/ preference/ difficult temperament | N= 121/292, 41%   1. No co-sleeping because sometimes that's all baby wants 2. Baby does not like to sleep alone- 3. Baby does not sleep in her cot. 4. no co-sleeping - baby would not settle unless sleeping on someone 5. Not bedsharing - it has been the easiest way for us both to get sleep, as baby wants to be close or cuddle while sleeping 6. Not sharing the same bed - He sleep better when he is close to me 7. Co-sleeping - there is now evidence to support proper co-sleeping and my baby spends large portions of each night sleeping on my chest 8. Placing baby on back. Often he wiggles about possibly wind when placed on back. He sleeps better on me as soon as I put him down he wakes 9. Sleep in bassinet - we bed share as baby needed skin to skin to settle 10. Co sleeping as my baby refuses to sleep away from me so I am up all night and cannot function the next day 11. Cosleep-it was only way to settle baby and so I could get sleep 12. sometimes baby is congested or windy and wants to sleep on me upright 13. Co sleeping as he is more calm when with me 14. Not having the baby sleep on you - this is the only position he would settle in on my chest 15. My baby would not sleep on his back for the first 4 weeks so i to hold her 16. separate surface - sometimes baby won't sleep except if feed laying down together 17. Do not have baby on sleeping parent's chest - baby will sleep in this position and it's difficult to stay awake sometimes 18. Cosleeping, my babies both are/were clingy 19. Sleeping with baby- Because she wouldn't settle and I was so tired 20. when she was first born, sleeping her in her own bed was difficult as she would not settle 21. Baby wanted to sleep on my chest, so had to persevere with bassinet training 22. no co-sleeping. this settles our baby and we follow advice on safe co-sleeping 23. it was hard not to sleep with baby for a couple of nights as she was up every 2hours 24. No co-sleeping - she would not settle in either cot or bassinet, despite being breastfed and falling asleep on breast 25. baby likes to be held almost all the time takes awhile to settle on own bed results in good sleep for the baby but poor sleep for mum 26. Co-sleeping - Sometimes he was unsettled but fell asleep when next to me in bed. 27. sleep baby in cot - our baby has lactose intolerance the only way we could get him to sleep at all was lying on his mothers chest. he has formed bad habits.. 28. sleeping in cot alone - because baby does not sleep well alone in cot, cries non-stop. parents don't get sleep as a result, but sleep well with parents in bed 29. no co-sleeping - sometimes the baby will only sleep close to me or on my chest. I fall asleep often 30. Sleeping alone with a needy toddler 31. don't co-sleep - baby would only sleep with me for first few 32. sleep in separate bed is hard because baby want to be close to you 33. sleep baby on separate surface - bassinet/cot. Baby constantly wakes and takes longer to re-settle 34. baby sleeping separately - too frequent wake up at night hence I get very exhausted 35. No co-sleeping has not really worked for me, my babies sleep better on my chest at first 36. baby sleeps better in our bed with us than on his own which means better sleep for all of us 37. no co-sleeping - my baby needs to feel me close to sleep well and I need sleep to be a good mother 38. Not co-sleeping because he likes body warmth and helps with my mental stability 39. co-sleeping- bub likes to sleep with me in my bed 40. Placing baby in own bed (sleep better with parents) 41. my baby likes to go to sleep with me beside him laying down with him then I put him in his bed 42. no co-sleeping - my baby finds it hard to stay asleep by themslef 43. Not now but in first 2months he would sleep in bed with me - safely however he wouldn't sleep alone especially while breastfeeding difficult staying awake! I breastfed in bed. 44. For baby to sleep in own sleeping area, she wakes if not touching my husband or I. 45. co-sleeping - even though this isn't recommended we have found this safest and settles baby best 46. 'safe sleeping environemnt' is not always easy to ahdere to as most/all very young babies will only settle on their mothers chest. 47. I co-sleep with baby - she won't sleep flat on back or side (need pillow) or on her belly 48. my baby will not sleep alone and needs to co-sleep occasionally 49. being in cot all the time as wont always settle on own 50. No co-sleeping, baby was very clingy and constantly woken by older sibling's noise 51. Not to co-sleep, because babies like to sleep with their mums 52. does't like to sleep alone - baby hates cot 53. I co-sleep with my baby often as she settles best this way and stays asleep longer. 54. co-sleeping - when newborn is hard to settle in cot/bassinet but sleeps well on mum 55. sleeping baby somewhere that isn't beside me eg. bassinet/cot - because he wont sleep without me beside him. He just cry non-stop and only sleeps all night if co-sleeping 56. initially baby liked being upright (possible reflux) on me. We slept in recliner with blanket tucking us is. Approx 4 weeks. 57. Baby only happily sleeps while being held 58. No co-sleeping- baby was very upset and won't sleep alone 59. My baby doesn't like to sleep in seperate bed after several trials 60. baby sleeping in cot on back - reflux baby, difficulty sleeping flat on back or in cot/bassinet, very unsettled, would only sleep upright with parent/grandparent 61. baby finds it hard to sleep in cot 62. when baby will only sleep on my chest and unable to put him in his cot as he will wake 63. Baby sleeps with us as he wont sleep in cot 64. Having baby in own bed, as they prefer closeness and being propped up 65. Sleeping in the cot- my baby likes to sleep on my side 66. Baby likes to sleep on side in bed with me- follow all other recommendations eg no blanket etc/no smoking 67. My baby currently shares my bed- He will not settle in his cot 68. no co-sleeping - he wants to sleep by me. He knows no different. Centuries ago mums wouldn't have had their babies away from them to sleep I don't know. Not natural. 69. Baby in cot/no co-sleeping because baby won't sleep anywhere else in the beginning 70. Co-sleeping. In early weeks baby will only sleep whilst being close or held. Co-sleeping was only way i could get any sleep 71. Baby to sleep on own sleep surface- she would only sleep 20 minutes max alone. 72. babies feel safe and secure within close proximity of their parents, they sleep better when they sleep with their parent(s) and you can breast feed them through the night much easier 73. Difficult not to co-sleep with baby as baby wakes constantly in bassinet but sleeps soundly when in bed with mother 74. sleep in own bed - as a newborn the only way baby would settle was on top of me. Sitting up on a couch for hours wasn't a safe option so she came to bed 75. Co-sleeping - sometimes it's only way he'll go to sleep 76. Sometimes it is difficult to get baby to sleep on their own sleeping surface. This is because when baby is unsettled they will keep waking when placed in bassinet. 77. co-sleeping, because during his 4 month regression he is awake every hour and he only settles in the early morning when i eventually put him in bed with me after his dad has gone to work. He also sleeps better in our king bed rather than his bassinet. 78. Sleep in bassinet cot as baby would only sleep in my arms in the day 79. not sleep with you - baby would not settle on own 80. Sleep in cot/ bassinet 100% of time - my sleep deprivation/ constant breastfeeding and baby that would not settle in cot, at times, resulted in some co-sleeping. Also it felt quiet natural to have baby close. 81. Baby liked to/ slept better on his stomach so he spent some nights sleeping up on my chest or being held. 82. we co-slept 2-11 weeks because she wouldn't sleep once she was put down. 83. no co-sleeping - my baby and I sleep much better when we co-sleep. He wakes up every 2 hours or sometimes more often to breastfeed and it is impossible for me to get rest if I have to get up from my bed to feed him every time. 84. co-sleeping - doesn't settle sometimes unless in bed with me. Practice safe co-sleeping 85. co-sleeping - baby became used to sleeping in parental bed during my partners job transition. She doesn't tolerate her own crib well since then (10weeks age onwards). Also it's easier to breastfeed at night when she is co-sleeping with us. 86. co-sleep - baby wont sleep on own surface 87. making baby sleep alone took too long to sleep 88. Baby in cot alone - when she is crying and wont go back to sleep (after a long time, e.g 4 hours!) baby co-sleeping/bed-sharing seems to be the only way she will sleep. 89. sleeping in own bed because baby wouldn't sleep unless cuddled 90. co-sleeping - it was hard when my baby wouldn't settle and just wanted to be with me so I made the co-sleeping space a safe space 91. sleep in own cot- baby does not settle back in own cot for 2nd half of night. co-sleep and breastfeed sevral hours of the morning. Very difficult when up several times feeding and being very sleep deprived 92. baby sleeps best beside mum so for parts of each night we co-sleep as of the last week due to 4 month sleep regression 93. avoiding cosleeping is difficult because there are times when my baby just wants to be close to me and I'm so exhausted from holding and/or rocking him 94. avoiding co-sleeping - early on it was difficult to make it through an entire night as she wouldn't settle unless on chest 95. Baby will not settle when place in bassinet/cot/pram. Baby likes to be constantly held. 96. co-sleeping - baby would not sleep unless held for first 4-5 weeks 97. baby sleeping on back - for the first 6 weeks she slept with me in bed on my chest so we as a family could get sleep, otherwise I would have been a danger to everyone. 98. no co-sleeping - sometimes its the only way she'd settle 99. no co-sleeping - baby has reflux and had colic. would not stop crying unless on us 100. co-sleeping in early day when not used to night waking and baby only settling on me. 101. Not co-sleeping- Sometimes baby just wants to be close 102. co sleeping in early weeks - My baby always woke when taken off my chest 103. Co-sleeping - because he wont sleep without me 104. No co-sleeping/ Baby sleeping alone- baby absolutely would not settle unless held skin to skin 105. Not sleeping with baby, first few weeks they sleep easy with mum and you are so tired that it is tempting to both get some sleep 106. do not co-sleep - baby sleeps longer and better when held or in bed with mother 107. when baby upset they sleep much longer and better when with me in bed instead of cot by themselves 108. bedsharing, baby settles better in our bed, easier to feed in the night. Was easier after a c-section 109. Co-sleeping - All my babies settle better lying next to me and night feeding is super easy 110. Baby in bed. baby has reflux and initially would only sleep upright to help I sat up and 'slept' holding her. 111. the only difficulty was when we were exhausted and putting baby to sleep by patting against chest and accidentally dosed off in bed with baby in arms (against chest). Baby often wouldn't settle if wasn't put to sleep without patting though. 112. sleeping alone. He wants to have a hand on his tummy or hold hand all night. Day sleeps he is fine alone. 113. baby wouldn't settle unless in arms - cried all night alone in cot, ended up co-sleeping for first few weeks. 114. baby sleeping in own bed as he wakes multiple times at night to feed (4-6times) 115. bedsharing & couch sleeping - he wouldn't sleep without being on us until 6 weeks 116. putting baby in a different room - he could only sleep with us 117. Co-sleeping - Baby spent first 8 weeks only wanting to sleep on my chest 118. very clingy baby and tired mummy - co-sleeping in a bed 119. avoiding co-sleeping - sometimes baby will only nap in contact with mum and mum gotta sleep sometime! 120. She has also slept in our bed on occasion (not regularly). It is difficult because you are tired and you do what works to get them to sleep! 121. sleep baby in cot/bassinet - when baby was little he would only sleep in our arms or in bed with us |
| Breastfeeding + feeding | N= 80/292, 27%   1. Co sleep when tired baby not settling or falling asleep when breastfeeding at night 2. Not falling asleep while feeding baby 3. Don't co-sleep: I get sleepy breastfeeding 4. Co sleeping - in the middle of the night doing lie down feeding, sometimes both mum and bub fall asleep inadvertently 5. separate surface - sometimes baby won't sleep except if feed laying down together 6. Not co sleeping difficult when breastfeeding 7. Not cosleeping. When exclusively BF it is better to plan to cosleep than to fall asleep feeding in a chair. 8. Independent bed - we feed in bed at night and tend to co-sleep after feeds 9. No co-sleeping. When completely exhausted I would bring baby into bed for a morning breastfeed/sleep 10. Con sleeping because I like having her next to me during the night. Plus more convenient with feeding. 11. not co-sleeping is difficult when breastfeeding and early morning feed, or when bubba is very unsettled 12. I do feed sitting in bed at night 13. in own bed - sleep while feeding 14. breastfeeding in bed with bub 15. not having baby in bed when breastfeeding 16. Not now but in first 2months he would sleep in bed with me - safely however he wouldn't sleep alone espeically while breastfeeding difficult staying awake! I breastfed in bed. 17. Co-sleep at times while breastfeeding 18. Baby sleeping in our bed, she settles better and easier in breastfeeding 19. sleeping in cot/bassinet wouldn't settle. we have a king bed so when bub was a bit older he slept there and settled and we all got more sleep especially with breastfeeding 20. staying awake and alert when taking for night feeds 21. having my child in his own bassinet for all sleep, because when breastfeeding I found it easier laying in bed with my son 22. I did struggle in first month putting bub back to bed. Would fall asleep with him feeding so he would sleep on me on his tummy for part of night 23. Sleeping in own environment- Easy to fall asleep together in bed at night feeds 24. i find the most difficult recommendation is to not sleep with baby in the same bed because I am breastfeeding my baby and she gets hungry easily. it is hard for me to keep moving her from bed to bed. 25. baby in own bed - baby and I enjoy co-sleeping especially for night time feeds but acknowledge this can be dangerous 26. not having baby in bed at the beginning. physical injuries from birth prevented easy mobility of mother and it was difficult to get up every 2 hours at night to feed. 27. co-sleeping - its so much easier to get an hour or two sleep when you sleep with her, especially in first 6 weeks and breastfeeding 28. co-sleeping - only when very unsettled and trying to breastfeed. I don't use a blanket on me when she is in bed and use arm to block her from my pillow or moving. only very rarely co-sleep maybe 1hour at a time 29. not co-sleep, sometimes we all need sleep and it's more dangerous falling asleep while breastfeeding as you may drop the baby. 30. co-sleeping - tiredness of getting up all hours at night to feed vs sleep safely in same bed - when you have other kids and commitments in daytime 31. avoid co-sleeping - I prefer to co-sleep as it is easier to feed, I can monitor my baby and enjoy sleeping with her 32. avoid co-sleeping as sometimes after breastfeeding when he is on my chest to be burped we fall asleep together 33. Falling asleep on same surface during overnight feeds in the early weeks 34. Co-sleeping- Middle of the night feeds 35. I have 3 kids now and find it difficult to stay awake for night feeds. We co sleep as safely as possible i.e. No loose blankets, firm sleep surface, no smoking drinking etc 36. babies feel safe and secure within close proximity of their parents, they sleep better when they sleep with their parent(s) and you can breast feed them through the night much easier 37. Baby having their own sleep space. Because at night i fall asleep while feeding and I wake up panicked about the baby 38. No bed sharing. When you are so tired you fall asleep feeding 39. Is hard to stay awake while breastfeeding in the night, being too tired is very dangerous. 40. Not feeding in bed is very difficult with a newborn as they feed so often at night. 41. I found it difficult to stay awake during night feeds and putting my son back in his cot straight after the feed 42. It is hard not have the baby fall asleep next to me during night time nursing. Why? because I am tired and I fall asleep while he is nursing. 43. Do not co-sleep as I found it helpful with breastfeeding and settling 44. Own space - baby co-sleeps/feeds on demand overnight 45. Sleep in cot/ bassinet 100% of time - my sleep deprivation/ constant breastfeeding and baby that would not settle in cot, at times, resulted in some co-sleeping. Also it felt quiet natural to have baby close. 46. sleeping on back when co-sleeping as baby feeds and then I fall asleep before I can turn baby back. I was feeding upright but would fall asleep and wake with baby in my arms 47. co-sleeping in bed when breastfeeding 48. no co-sleeping - my baby and I sleep much better when we co-sleep. He wakes up every 2 hours or sometimes more often to breastfeed and it is impossible for me to get rest if I have to get up from my bed to feed him every time. 49. co-sleeping - baby became used to sleeping in parental bed during my partners job transition. She doesn't tolerate her own crib well since then (10weeks age onwards). Also it's easier to breastfeed at night when she is co-sleeping with us. 50. sometimes falling asleep when feeding meant baby was on his side 51. no co-sleep because I get too tired and fall asleep while feeding 52. my baby will sleep with me on occasion, sometimes she wants comfort and will cluster feed 53. Co-sleeping- I fall asleep breastfeeding with baby on my chest at night. 54. co-sleeping, while worried me, made life easier with breastfeeding during night after many nights with little or no sleep 55. also staying awake while feeding in early days 56. Not to sleep in same bed as co-sleeping helpful in early weeks to decrease crying and make breastfeeding easier 57. also no bed sharing - we bedshare now she is older, not all night, or everyday, but for short periods of unsettled behaviours and nursing early mornings 58. Breastfeeding in bed and falling asleep at night whilst holding baby. It is difficult when the baby is young and wakes a lot and I am tired. I unintentionally fall asleep also because of the cold weather, I'm too cold to sit out of bed through the night. 59. don't co-sleep. Hard not to fall asleep feeding in bed 60. sleep in own cot- baby does not settle back in own cot for 2nd half of night. co-sleep and breastfeed several hours of the morning. Very difficult when up several times feeding and being very sleep deprived 61. in own bassinet - I often fall asleep during night feeds. 62. not to co-sleep because as baby becomes bigger I get used to having them next to me in bed while feeding 63. no co-sleep - its hard to do night breastfeeding if baby is in the cot. I'll be deadly sleepy now. I co-sleep with baby and both of us love it. 64. Co-sleeping!- easy to feed in bed during the night and half sleep/nap whilst feeding 65. not bedsharing - difficult to resettle bub after feeds and put back in bassinet 66. co-sleeping avoidance is most difficult and I haven't avoided it all because bub and I sleep better and feeding is easier when co-sleeping 67. bedsharing, baby settles better in our bed, easier to feed in the night. Was easier after a c-section 68. no co-sleeping - especially difficult when breastfeeding in the first few weeks 69. sometimes it was really nice to put baby in bed with us for the last feed before everyone got up, and feed him in bed (4-5am feed) then everyone get up at 6am 70. Co-sleeping- sometimes I'm so tired during middle of the night feeds I will bring bub to bed and follow advice I was given from midwives. 71. Co-sleeping - All my babies settle better lying next to me and night feeding is super easy 72. Avoid co-sleeping - when sleep deprived it is easier to bring baby back to bed to feed & settle sometimes. 73. co-sleeping when really tired and breast-feeding in bed and mother falls asleep with baby still on boob 74. co-bedding particularly when overnight feeding and very fatigued 75. Own cot - for breastfeeding 76. no co-sleeping - its so much easier to feed at night when co-sleeping 77. Baby has own bed, because it’s so easy to fall asleep feeding when you’re sleep deprived. It seems natural to bedshare with a newborn 78. we co-sleep to feed at night 79. Night feeding- whilst breastfeeding its easier to accidentally fall asleep with baby. You're more alert if you need to make a bottle. 80. Sleep in crib when little - lots of feeds and very tiring getting up for every one. You wake up and hard to fall asleep. Bedshared about week 7-12. |
| Exhaustion/ Fatigue | N =77/292, 26%   1. Not co sleeping because you are so tired 2. Co sleep when tired baby not settling or falling asleep when breastfeeding at night 3. Not falling asleep while feeding baby 4. Don't co-sleep: I get sleepy breastfeeding 5. Co sleeping, too tired to put back to bed 6. Co sleeping as my baby refuses to sleep away from me so I am up all night and cannot function the next day- As I have another child I need to get sleep when I can especially at night 7. Falling asleep holding baby-if we are both comfortable I have 8. Co sleeping - in the middle of the night doing lie down feeding, sometimes both mum and bub fall asleep inadvertently 9. Do not have baby on sleeping parent's chest - baby will sleep in this position and it's difficult to stay awake sometimes 10. Sleeping with baby- Because she wouldn't settle and I was so tired 11. Not cosleeping. When exclusively BF it is better to plan to cosleep than to fall asleep feeding in a chair. 12. It's sometimes difficult to resist the temptation to sleep with baby in my bed with me for at least short periods, especially when baby sleeps better that way and just a few more minutes sleep that it would result in seems so valuable. 13. No co-sleeping. When completely exhausted I would bring baby into bed for a morning breastfeed/sleep 14. sleeping in cot alone - because baby does not sleep well alone in cot, cries non-stop. parents don't get sleep as a result, but sleep well with parents in bed 15. no co-sleeping - sometimes the baby will only sleep close to me or on my chest. I fall asleep often 16. Exhaustion will lead to having the baby in bed with you 17. Co-sleeping - He has reflux and doesn't sleep/settle very well. For me to get some sleep it's easier if he is in the same bed so I can settle him. 18. baby sleeping separately - too frequent wake up at night hence I get very exhausted 19. no co-sleeping - my baby needs to feel me close to sleep well and I need sleep to be a good mother 20. baby to have own sleeping space - baby has been unsettled, averaging 1-2hours sleep. midwife aware that baby has been sleeping with mum in bed due to exhaustion 21. Not now but in first 2months he would sleep in bed with me - safely however he wouldn't sleep alone especially while breastfeeding difficult staying awake! I breastfed in bed. 22. Also mums who are desperate for sleep will sometimes not follow safe sleeping recommendations for the sake of getting some sleep. 23. co sleep - sometimes you just do what you can to get rest 24. staying awake and alert when taking for night feeds 25. I did struggle in first month putting bub back to bed. Would fall asleep with him feeding so he would sleep on me on his tummy for part of night 26. co-sleeping - tiredness and need for baby to be close 27. co-sleeping - its so much easier to get an hour or two sleep when you sleep with her, especially in first 6 weeks and breastfeeding 28. not co-sleep, sometimes we all need sleep and it's more dangerous falling asleep while breastfeeding as you may drop the baby. 29. co-sleeping - tiredness of getting up all hours at night to feed vs sleep safely in same bed - when you have other kids and commitments in daytime 30. avoid co-sleeping as sometimes after breastfeeding when he is on my chest to be burped we fall asleep together 31. sometimes she sleeps in our bed because she is unsettled and we are tired 32. C-sleeping- exhaustion, however no blankets are used and baby's father sleeps in different bed. 33. Co-sleep - not by choice but sometimes exhaustion so I fell asleep 34. co sleep- it gets us both better sleep. 3rd baby need sleep 35. Falling asleep on same surface during overnight feeds in the early weeks 36. Co-sleeping. In early weeks baby will only sleep whilst being close or held. Co-sleeping was only way i could get any sleep 37. I have 3 kids now and find it difficult to stay awake for night feeds. We co sleep as safely as possible i.e. No loose blankets, firm sleep surface, no smoking drinking etc 38. Baby having their own sleep space. Because at night i fall asleep while feeding and I wake up panicked about the baby 39. No bed sharing. When you are so tired you fall asleep feeding 40. Is hard to stay awake while breastfeeding in the night, being too tired is very dangerous. 41. I found it difficult to stay awake during night feeds and putting my son back in his cot straight after the feed 42. It is hard not have the baby fall asleep next to me during night time nursing. Why? because I am tired and I fall asleep while he is nursing. 43. Baby not sleeping on parents while parents fall asleep at night time 44. Sometimes baby sleeps with me so that I can get some sleep too. 45. Sleep in cot/ bassinet 100% of time - my sleep deprivation/ constant breastfeeding and baby that would not settle in cot, at times, resulted in some co-sleeping. Also it felt quite natural to have baby close. 46. For me, only the last. For both my sons, they have ended up in my bed at some point in the night. If I didn't allow this, I am not sure how I would have gotten any sleep. 47. sleeping on back when co-sleeping as baby feeds and then I fall asleep before I can turn baby back. I was feeding upright but would fall asleep and wake with baby in my arms 48. often fall asleep with baby in my arms during the night, baby has reflux and is/was more comfortable upright on my tummy than lying down 49. sleep by themselves - as baby wakes every one to 2 hours 50. no co-sleep because I get too tired and fall asleep while feeding 51. Co-sleeping- I fall asleep breastfeeding with baby on my chest at night. 52. co-sleeping, while worried me, made life easier with breastfeeding during night after many nights with little or no sleep 53. Avoiding co-sleeping - Its the only way to get rest sometimes in first few months and is good for bonding. 54. also staying awake while feeding in early days 55. Breastfeeding in bed and falling asleep at night whilst holding baby. It is difficult when the baby is young and wakes a lot and I am tired. I unintentionally fall asleep also because of the cold weather, I'm too cold to sit out of bed through the night. 56. don't co-sleep. Hard not to fall asleep feeding in bed 57. sleep in own cot- baby does not settle back in own cot for 2nd half of night. co-sleep and breastfeed sevral hours of the morning. Very difficult when up several times feeding and being very sleep deprived 58. avoiding cosleeping is difficult because there are times when my baby just wants to be close to me and I'm so exhausted from holding and/or rocking him 59. co sleeping - when she was unsettled/ or I was tired she went to sleep beside me in bed 60. baby sleeping on back - for the first 6 weeks she slept with me in bed on my chest so we as a family could get sleep, otherwise I would have been a danger to everyone. 61. co-sleeping in early day when not used to night waking and baby only settling on me. 62. in own bassinet - I often fall asleep during night feeds. 63. it is easy to co-sleep especially when so sleep deprived 64. Not sleeping with baby, first few weeks they sleep easy with mum and you are so tired that it is tempting to both get some sleep 65. Occasionally day sleep with bub or bring bub into my bed at 5am- too tired. Toddler I have is a terrible sleeper and I need to sleep when I can 66. Co-sleeping- sometimes I'm so tired during middle of the night feeds I will bring bub to bed and follow advice I was given from midwives. 67. Avoid co-sleeping - when sleep deprived it is easier to bring baby back to bed to feed & settle sometimes. 68. the only difficulty was when we were exhausted and putting baby to sleep by patting against chest and accidentally dosed off in bed with baby in arms (against chest). Baby often wouldn't settle if wasn't put to sleep without patting though. 69. co-sleeping when really tired and breast-feeding in bed and mother falls asleep with baby still on boob 70. co-bedding particularly when overnight feeding and very fatigued 71. Baby has own bed, because it’s so easy to fall asleep feeding when you’re sleep deprived. It seems natural to bedshare with a newborn 72. very clingy baby and tired mummy - co-sleeping in a bed 73. avoiding co-sleeping - sometimes baby will only nap in contact with mum and mum gotta sleep sometime! 74. She has also slept in our bed on occasion (not regularly). It is difficult because you are tired and you do what works to get them to sleep! 75. Night feeding- whilst breastfeeding its easier to accidentally fall asleep with baby. You're more alert if you need to make a bottle. 76. Sleep in crib when little - lots of feeds and very tiring getting up for every one. You wake up and hard to fall asleep. Bedshared about week 7-12. 77. co-sleeping (for part of night) so I can get a little extra sleep from about 4 or 5 in the morning |
| Better/ more sleep (for mother and/ or baby) | N= 63/292, 22%   1. Not co-sleeping. Baby sleeps longer and easier to settle if co-sleeping 2. Not bedsharing - it has been the easiest way for us both to get sleep, as baby wants to be close or cuddle while sleeping 3. Not sharing the same bed - He sleep better when he is close to me 4. Placing baby on back. Often he wiggles about possibly wind when placed on back. He sleeps better on me as soon as I put him down he wakes 5. Co sleeping as my baby refuses to sleep away from me so I am up all night and cannot function the next day 6. Co sleep-it was only way to settle baby and so I could get sleep 7. separate surface - sometimes baby won't sleep except if feed laying down together 8. It's sometimes difficult to resist the temptation to sleep with baby in my bed with me for at least short periods, especially when baby sleeps better that way and just a few more minutes sleep that it would result in seems so valuable. 9. baby likes to be held almost all the time takes a while to settle on own bed results in good sleep for the baby but poor sleep for mum 10. own sleep surface - baby never sleeps as well alone 11. sleeping in cot alone - because baby does not sleep well alone in cot, cries non-stop. parents don't get sleep as a result, but sleep well with parents in bed 12. co-sleep sometimes at 4am my baby comes into bed with me as its the only way to get some sleep for both of us after 4am 13. Co-sleeping - He has reflux and doesn't sleep/settle very well. For me to get some sleep it's easier if he is in the same bed so I can settle him. 14. No co-sleeping has not really worked for me, my babies sleep better on my chest at first 15. baby sleeps better in our bed with us than on his own which means better sleep for all of us 16. no co-sleeping - my baby needs to feel me close to sleep well and I need sleep to be a good mother 17. Placing baby in own bed (sleep better with parents) 18. Not to sleep with baby, it is convenient and baby sleeps better 19. sleeping in cot/bassinet wouldn't settle. we have a king bed so when bub was a bit older he slept there and settled and we all got more sleep especially with breastfeeding 20. co sleep - sometimes you just do what you can to get rest 21. I co-sleep with my baby often as she settles best this way and stays asleep longer. 22. co-sleeping - when newborn is hard to settle in cot/bassinet but sleeps well on mum 23. sleeping baby somewhere that isn't beside me eg. bassinet/cot - because he wont sleep without me beside him. He just cry non-stop and only sleeps all night if co-sleeping 24. co-sleeping - its so much easier to get an hour or two sleep when you sleep with her, especially in first 6 weeks and breastfeeding 25. sleeping in cot is difficult - baby used to sleep in her cot but she rarely slept tight. She now sleeps in the same bed with us and can sleep so much better 26. own sleeping area- sometimes we co-sleep to ensure we both (baby and mother) get sleep 27. co-sleeping - tiredness of getting up all hours at night to feed vs sleep safely in same bed - when you have other kids and commitments in daytime 28. sometimes easier and baby sleeps better if we co-sleep 29. co sleep- it gets us both better sleep. 3rd baby need sleep 30. own sleep surface. It's much easier to get them to sleep later/longer in parental bed 31. Cosleeping because it is only thing that works sometimes 32. Co-sleeping. In early weeks baby will only sleep whilst being close or held. Co-sleeping was only way i could get any sleep 33. Baby to sleep on own sleep surface- she would only sleep 20 minutes max alone. 34. babies feel safe and secure within close proximity of their parents, they sleep better when they sleep with their parent(s) and you can breast feed them through the night much easier 35. Difficult not to co-sleep with baby as baby wakes constantly in bassinet but sleeps soundly when in bed with mother 36. Sometimes baby sleeps with me so that I can get some sleep too. 37. baby to sleep on their own. at the moment baby sleeps better with me in bed in the early hours of the morning 38. co-sleeping, because during his 4 month regression he is awake every hour and he only settles in the early morning when i eventually put him in bed with me after his dad has gone to work. He also sleeps better in our king bed rather than his bassinet. 39. For me, only the last. For both my sons, they have ended up in my bed at some point in the night. If I didn't allow this, I am not sure how I would have gotten any sleep. 40. no co-sleeping - my baby and I sleep much better when we co-sleep. He wakes up every 2 hours or sometimes more often to breastfeed and it is impossible for me to get rest if I have to get up from my bed to feed him every time. 41. own surface in same room - he generally sleeps in cradle for start of night then in to co-sleep with me - we get a lot more sleep and feel half human! 42. co-sleeping, while worried me, made life easier with breastfeeding during night after many nights with little or no sleep 43. Avoiding co-sleeping - Its the only way to get rest sometimes in first few months and is good for bonding. 44. when baby wakes so frequently having them in your bed helps mother and baby get back to sleep faster 45. no cosleeping - my baby sleeps better with me sometimes 46. Baby in cot alone - when she is crying and wont go back to sleep (after a long time, e.g 4 hours!) baby co-sleeping/bed-sharing seems to be the only way she will sleep. 47. sleeping in own bed because baby wouldn't sleep unless cuddled 48. baby sleeps best beside mum so for parts of each night we co-sleep as of the last week due to 4 month sleep regression 49. sleep on a separate surface - we both sleep well when we co-sleep 50. baby sleeping on back - for the first 6 weeks she slept with me in bed on my chest so we as a family could get sleep, otherwise I would have been a danger to everyone. 51. Not sleep with baby as they sleep better 52. sleep on a separate surface - we both sleep well when we co-sleep 53. Not sleeping with baby, first few weeks they sleep easy with mum and you are so tired that it is tempting to both get some sleep 54. Occasionally day sleep with bub or bring bub into my bed at 5am- too tired. Toddler I have is a terrible sleeper and I need to sleep when I can 55. do not co-sleep - baby sleeps longer and better when held or in bed with mother 56. We had to co sleep, have cot with side off attached to bed so that we could get some sleep. Baby goes back in cot after feeds 57. co-sleeping avoidance is most difficult and I haven't avoided it all because bub and I sleep better and feeding is easier when co-sleeping 58. we co-slept often when we first came home as he slept longer - I believe you do not tend to go into a deep sleep when you know they are there. 59. when baby upset they sleep much longer and better when with me in bed instead of cot by themselves 60. I find it hard to let the baby sleep in their own place because she wont sleep for long hours 61. Sleep on own bed. Our baby co-sleeps, she and I both get more sleep this way. 62. Sleep in crib when little - lots of feeds and very tiring getting up for everyone. You wake up and hard to fall asleep. Bedshared about week 7-12. 63. co-sleeping (for part of night) so I can get a little extra sleep from about 4 or 5 in the morning |
| Comforting for infants (soothing, settling)  Comforting for mother/ parent--parental preference/ enjoyment/ satisfaction/ pride/ comfort in bed-sharing | N= 62/292, 21%   1. Baby in cot. I believe in co-sleeping. I do not want to be separate from baby. 2. Co sleep when tired baby not settling or falling asleep when breastfeeding at night 3. co-sleeping as baby would only settle when on my chest 4. Sometimes I have slept on the floor in his room on a blanket with him swaddled next to me if he was particularly unsettled 5. Not co-sleeping. Baby sleeps longer and easier to settle if co-sleeping 6. no co-sleeping - baby would not settle unless sleeping on someone 7. Not bedsharing - it has been the easiest way for us both to get sleep, as baby wants to be close or cuddle while sleeping 8. Sleep alone, prefer co-sleeping 9. Sleep in bassinet - we bed share as baby needed skin to skin to settle 10. sometimes baby is congested or windy and wants to sleep on me upright 11. Co sleeping as he is more calm when with me 12. Not having the baby sleep on you - this is the only position he would settle in on my chest 13. Cosleeping, my babies both are/were clingy 14. Co-sleeping initially helped me settle bub in the first month 15. Sleeping with baby- Because she wouldn't settle and I was so tired 16. when she was first born, sleeping her in her own bed was difficult as she would not settle 17. Baby settles easier with cosleeping 18. No cosleeping. 95% of the time is in her own bassinet, but sometimes when wouldn't settle found it difficult not to put her on my chest to sleep 19. Con sleeping because I like having her next to me during the night. Plus more convenient with feeding. 20. Co-sleeping - Sometimes he was unsettled but fell asleep when next to me in bed. 21. We do allow the baby in bed area though co-sleeping is not advised. I have read widely on it and know the safest positions etc. I feel like it comforts the baby (although he sleeps fine in the bassinet) 22. I prefer co-sleeping 23. We used a baby sleep nest (cocoonababy) to help him settle in the first 3 months - so 'cot only' was difficult 24. Not co-sleeping because he likes body warmth and helps with my mental stability 25. no co-sleeping in same bed when baby will not settle! 26. Co-sleeping I believe is a form of bonding with your baby, my baby settles better in my bed 27. Baby sleeping in our bed, she settles better and easier in breastfeeding 28. I co-sleep with my baby often as she settles best this way and stays asleep longer. 29. co-sleeping - when newborn is hard to settle in cot/bassinet but sleeps well on mum 30. sleeping baby somewhere that isn't beside me eg. bassinet/cot - because he won’t sleep without me beside him. He just cry non-stop and only sleeps all night if co-sleeping 31. baby in own bed - baby and I enjoy co-sleeping especially for night time feeds but acknowledge this can be dangerous 32. co-sleeping - tiredness and need for baby to be close 33. At first baby settled better co-sleeping but has now got used to bassinet 34. sleep baby on back - is difficult because baby sleeps in bed with me and often falls asleep while feeding on her side 35. Co-sleeping- Dad wanted baby in middle of us both 36. avoid co-sleeping - I prefer to co-sleep as it is easier to feed, I can monitor my baby and enjoy sleeping with her 37. I liked co-sleeping with my baby 38. Do not share sleeping surface with baby as it is sometimes easier to settle him in my bed 39. Sleep in cot - was fine when he was newborn but now it is better he is in bed with me 40. Co sleeping because it helps to settle baby 41. co-sleeping, because during his 4 month regression he is awake every hour and he only settles in the early morning when I eventually put him in bed with me after his dad has gone to work. He also sleeps better in our king bed rather than his bassinet. 42. Do not co-sleep as I found it helpful with breastfeeding and settling 43. Sleep in cot/ bassinet 100% of time - my sleep deprivation/ constant breastfeeding and baby that would not settle in cot, at times, resulted in some co-sleeping. Also it felt quite natural to have baby close. 44. no bedsharing - greatly under estimated for health benefits of bub and mother. most natural method on the planet. safe sleeping should be taught for bed sharing 45. co-sleeping - doesn't settle sometimes unless in bed with me. Practice safe co-sleeping 46. sick baby - they only want mum so easy to lay with baby while sick 47. my baby will sleep with me on occasion, sometimes she wants comfort and will cluster feed 48. Not sleeping in the big bed- we often fall asleep watching TV or cuddling in bed. 49. often the baby wont settle when he's sick with a cold. I have had to sleep with him in my bed more often to help him settle and breathe. 50. Baby in cot alone - when she is crying and wont go back to sleep (after a long time, e.g 4 hours!) baby co-sleeping/bed-sharing seems to be the only way she will sleep. 51. not sleep with bub - I think natural to cuddle and sleep with baby. Husband African - they all sleep with baby there. Baby stops crying and breathes better when I snuggle him. 52. Not to co-sleep is difficult in the first few days/weeks as being close is comforting 53. not to co-sleep because as baby becomes bigger I get used to having them next to me in bed while feeding 54. no co-sleep - its hard to do night breastfeeding if baby is in the cot. I'll be deadly sleepy now. I co-sleep with baby and both of us love it. 55. no co-sleeping - because its so lovely to cuddle up with baby and also very close by to attend to 56. Putting baby in cot. Baby will scream and I feel insecure leaving my baby in a sterile cot which resembles a jail. We are the only mammals who sleep away from our young. It is tragic. 57. sometimes it was really nice to put baby in bed with us for the last feed before everyone got up, and feed him in bed (4-5am feed) then everyone get up at 6am 58. Baby co-sleeps, we are both most comfortable this way. 59. Co-sleeping - All my babies settle better lying next to me and night feeding is super easy 60. sleeping alone. He wants to have a hand on his tummy or hold hand all night. Day sleeps he is fine alone. 61. baby wouldn't settle unless in arms - cried all night alone in cot, ended up co-sleeping for first few weeks. 62. not co-sleeping - as it is easier for both of us to sleep when he's in bed with me |
| Crying (unsettled baby) | N= 58/292, 20%   1. Do not co-sleep- have had extremely windy baby who struggles to settle. Have brought baby into bed for a couple of hours here and there very occasionally 2. No cosleeping. 95% of the time is in her own bassinet, but sometimes when wouldn't settle found it difficult not to put her on my chest to sleep 3. Haven't always used to cot - practice co-sleeping sometimes if he is very unsettled but follow guidelines for safest way to co-sleep 4. it was hard not to sleep with baby for a couple of nights as she was up every 2hours 5. sleep baby in cot - our baby has lactose intolerance the only way we could get him to sleep at all was lying on his mothers chest. he has formed bad habits. 6. sleeping in cot alone - because baby does not sleep well alone in cot, cries non-stop. parents don't get sleep as a result, but sleep well with parents in bed 7. not co-sleeping is difficult when breastfeeding and early morning feed, or when bubba is very unsettled 8. sometimes sleep with baby on my chest when wont settle 9. Co-sleeping - He has reflux and doesn't sleep/settle very well. For me to get some sleep it's easier if he is in the same bed so I can settle him. 10. no co-sleeping in same bed when baby will not settle! 11. baby to have own sleeping space - baby has been unsettled, averaging 1-2hours sleep. midwife aware that baby has been sleeping with mum in bed due to exhaustion 12. sleeping in cot/bassinet wouldn't settle. we have a king bed so when bub was a bit older he slept there and settled and we all got more sleep especially with breastfeeding 13. 'safe sleeping environemnt' is not always easy to ahdere to as most/all very young babies will only settle on their mothers chest. 14. when baby was <3weeks, would not sleep lying flat - slept holding her upright on lounge. Baby did not like her cot - not comfortable or pleasant with just a fitted sheet & blanket. 15. sleeping baby somewhere that isn't beside me eg. bassinet/cot - because he wont sleep without me beside him. He just cry non-stop and only sleeps all night if co-sleeping 16. initially baby liked being upright (possible reflux) on me. We slept in recliner with blanket tucking us is. Approx 4 weeks. 17. No co-sleeping- baby was very upset and won't sleep alone 18. Unless baby unsettled at night, is easier to co-sleep but try not to 19. baby sleeping in cot on back - reflux baby, difficulty sleeping flat on back or in cot/bassinet, very unsettled, would only sleep upright with parent/grandparent 20. no co-sleeping, when baby unsettled especially the first few weeks 21. sometimes I co-sleep with baby if she is fussing, not often. When I have to I try to sit up and have her sleep on my chest 22. Sleep in own bed - both children never settled - using clip on co-sleeping bay (baby bay) 23. Sleeping in bassinet/cot - can be challenging with unsettled baby 24. Co-sleeping when baby unsettled 25. co-sleeping - only when very unsettled and trying to breastfeed. I don't use a blanket on me when she is in bed and use arm to block her from my pillow or moving. only very rarely co-sleep maybe 1hour at a time 26. sometimes she sleeps in our bed because she is unsettled and we are tired 27. Sometimes co-sleeping seems tempting as they get bigger/not sleeping well 28. Avoid sharing a sleeping space- When baby has wind or reflux she only sleeps when held 29. Getting baby down to sleep in the bassinet. Due to baby already having had the flu, he slept in the bed with us in our king size bed in a controlled environment on a sleeping pad with walls for a long time and now is restless and upset in the bassinet. 30. sleep in own bed - as a newborn the only way baby would settle was on top of me. Sitting up on a couch for hours wasn't a safe option so she came to bed 31. Sometimes it is difficult to get baby to sleep on their own sleeping surface. This is because when baby is unsettled, they will keep waking when placed in bassinet. 32. co-sleeping, because during his 4 month regression he is awake every hour and he only settles in the early morning when i eventually put him in bed with me after his dad has gone to work. He also sleeps better in our king bed rather than his bassinet. 33. co-sleeping due to unsettled baby in first 10 weeks (finally diagnosed with silent reflux) 34. i often fall asleep with baby in my arms during the night, baby has reflux and is/was more comfortable upright on my tummy than lying down 35. sick baby - they only want mum so easy to lay with baby while sick 36. Our baby wont sleep anywhere other than with us. He suffers from bad wind all night. 37. often the baby wont settle when he's sick with a cold. I have had to sleep with him in my bed more often to help him settle and breathe. 38. Not to sleep in same bed as co-sleeping helpful in early weeks to decrease crying and make breastfeeding easier 39. also no bed sharing - we bedshare now she is older, not all night, or everyday, but for short periods of unsettled behaviours and nursing early mornings 40. Baby in cot alone - when she is crying and wont go back to sleep (after a long time, e.g 4 hours!) baby co-sleeping/bed-sharing seems to be the only way she will sleep. 41. co-sleeping - it was hard when my baby wouldn't settle and just wanted to be with me so I made the co-sleeping space a safe space 42. co sleeping - when she was unsettled/ or I was tired she went to sleep beside me in bed 43. not sleep with bub - I think natural to cuddle and sleep with baby. Husband African - they all sleep with baby there. Baby stops crying and breathes better when I snuggle him. 44. avoiding co-sleeping - early on it was difficult to make it through an entire night as she wouldn't settle unless on chest 45. no co-sleeping - sometimes its the only way she'd settle 46. no co-sleeping - baby has reflux and had colic. would not stop crying unless on us 47. co-sleeping in early day when not used to night waking and baby only settling on me. 48. sleeping in bassinet - unable to settle 49. sleeping with baby - when baby really unsettled, on occasions this was the only solution (slept with baby on higher slope end of couch seat so I would roll away from him) 50. No co-sleeping/ Baby sleeping alone- baby absolutely would not settle unless held skin to skin 51. not bedsharing - difficult to resettle bub after feeds and put back in bassinet 52. when baby upset they sleep much longer and better when with me in bed instead of cot by themselves 53. Putting baby in cot. Baby will scream and I feel insecure leaving my baby in a sterile cot which resembles a jail. We are the only mammals who sleep away from our young. It is tragic. 54. Baby in bed. baby has reflux and initially would only sleep upright to help I sat up and 'slept' holding her. 55. Avoid co-sleeping - when sleep deprived it is easier to bring baby back to bed to feed & settle sometimes. 56. the only difficulty was when we were exhausted and putting baby to sleep by patting against chest and accidentally dosed off in bed with baby in arms (against chest). Baby often wouldn't settle if wasn't put to sleep without patting though. 57. baby wouldn't settle unless in arms - cried all night alone in cot, ended up co-sleeping for first few weeks. 58. I find it hard not to co-sleep with baby when she is really unsettled. |
| Convenience/ease | N= 30/292, 10%   1. Not bedsharing - it has been the easiest way for us both to get sleep, as baby wants to be close or cuddle while sleeping 2. Baby settles easier with cosleeping 3. Con sleeping because I like having her next to me during the night. Plus more convenient with feeding. 4. Not to sleep with baby, it is convenient and baby sleeps better 5. Baby sleeping in our bed, she settles better and easier in breastfeeding 6. Sleeping in thiner own bed, because I had a c-section it was easier for baby to sleep in our bed because it was too painful to get out of bed 7. having my child in his own bassinet for all sleep, because when breastfeeding I found it easier laying in bed with my son 8. Sleeping in own environment- Easy to fall asleep together in bed at night feeds 9. i find the most difficult recommendation is to not sleep with baby in the same bed because I am breastfeeding my baby and she gets hungry easily. it is hard for me to keep moving her from bed to bed. 10. not having baby in bed at the beginning. physical injuries from birth prevented easy mobility of mother and it was difficult to get up every 2 hours at night to feed. 11. avoid co-sleeping - I prefer to co-sleep as it is easier to feed, I can monitor my baby and enjoy sleeping with her 12. sometimes easier and baby sleeps better if we co-sleep 13. Put baby to sleep in its own place - difficult to get up to baby after a c-section, easier for it to stay in bed with me through the night 14. SLEEP IN COT BECAUSE MOTHER HAD A C SECTION AND THE WOUND MADE IT DIFFICULT TO GET UP FROM THE BED TO THE COT 15. own sleep surface. It's much easier to get them to sleep later/longer in parental bed 16. Do not share sleeping surface with baby as it is sometimes easier to settle him in my bed 17. babies feel safe and secure within close proximity of their parents, they sleep better when they sleep with their parent(s) and you can breast feed them through the night much easier 18. Never co sleeping especially when baby is newborn its easier to have them next to you 19. co-sleeping - baby became used to sleeping in parental bed during my partners job transition. She doesn't tolerate her own crib well since then (10weeks age onwards). Also it's easier to breastfeed at night when she is co-sleeping with us. 20. Not to sleep in same bed as co-sleeping helpful in early weeks to decrease crying and make breastfeeding easier 21. Sometimes with newborn co-sleeping is easiest 22. it is easy to co-sleep especially when so sleep deprived 23. Not sleeping with baby, first few weeks they sleep easy with mum and you are so tired that it is tempting to both get some sleep 24. Co-sleeping!- easy to feed in bed during the night and half sleep/nap whilst feeding 25. co-sleeping avoidance is most difficult and I haven't avoided it all because bub and I sleep better and feeding is easier when co-sleeping 26. bedsharing, baby settles better in our bed, easier to feed in the night. Was easier after a c-section 27. Co-sleeping - All my babies settle better lying next to me and night feeding is super easy 28. Avoid co-sleeping - when sleep deprived it is easier to bring baby back to bed to feed & settle sometimes. 29. not co-sleeping - as it is easier for both of us to sleep when he's in bed with me 30. no co-sleeping - its so much easier to feed at night when co-sleeping |
| *Maternal instinct/ cosleeping is natural | N= 7/292, 2%   1. no co-sleeping - he wants to sleep by me. He knows no different. Centuries ago mums wouldn't have had their babies away from them to sleep I don't know. Not natural. 2. co sleeping - why not when asian families co sleep it makes no sense after 9 months in my belly to be seperated from me. 3. Sleep in cot/ bassinet 100% of time - my sleep deprivation/ constant breastfeeding and baby that would not settle in cot, at times, resulted in some co-sleeping. Also it felt quite natural to have baby close. 4. no bedsharing - greatly under estimated for health benefits of bub and mother. most natural method on the planet. safe sleeping should be taught for bed sharing 5. not sleep with bub - I think natural to cuddle and sleep with baby. Husband African - they all sleep with baby there. Baby stops crying and breathes better when I snuggle him. 6. Putting baby in cot. Baby will scream and I feel insecure leaving my baby in a sterile cot which resembles a jail. We are the only mammals who sleep away from our young. It is tragic. 7. Baby has own bed, because it’s so easy to fall asleep feeding when you’re sleep deprived. It seems natural to bedshare with a newborn |
| Monitoring/safety/ protection | N= 6/292, 2%   1. No co-sleeping. Our room is not large enough to have a cot or bassinet and I prefer to have our baby sleep with us so I can monitor her closely at all times 2. co-sleeping - even though this isn't recommended we have found this safest and settles baby best 3. avoid co-sleeping - I prefer to co-sleep as it is easier to feed, I can monitor my baby and enjoy sleeping with her 4. Co-sleeping- My sister passed from SIDS and I find it difficult to not have my baby next to me in case she stops breathing 5. no co-sleeping - because its so lovely to cuddle up with baby and also very close by to attend to 6. Putting baby in cot. Baby will scream and I feel insecure leaving my baby in a sterile cot which resembles a jail. We are the only mammals who sleep away from our young. It is tragic. |
| Needed due to (Mother’s) injury/ caesarean | N= 6/292, 2%   1. Sleeping in thiner own bed, because I had a c-section it was easier for baby to sleep in our bed because it was too painful to get out of bed 2. not having baby in bed at the beginning. physical injuries from birth prevented easy mobility of mother and it was difficult to get up every 2 hours at night to feed. 3. Put baby to sleep in its own place - difficult to get up to baby after a c-section, easier for it to stay in bed with me through the night 4. SLEEP IN COT BECAUSE MOTHER HAD A C SECTION AND THE WOUND MADE IT DIFFICULT TO GET UP FROM THE BED TO THE COT 5. Breastfeeding at night after a c-section. My partner works away & wasn't able to assist so I often co slept 6. bedsharing, baby settles better in our bed, easier to feed in the night. Was easier after a c-section |
| *Disagree with danger | N= 5/292, 2%   1. Co-sleeping - there is now evidence to support proper co-sleeping and my baby spends large portions of each night sleeping on my chest 2. co-sleeping - even though this isn't recommended we have found this safest and settles baby best 3. co sleeping - why not when asian families co sleep it makes no sense after 9 months in my belly to be seperated from me. 4. no bedsharing - greatly under estimated for health benefits of bub and mother. most natural method on the planet. safe sleeping should be taught for bed sharing 5. we co-slept often when we first came home as he slept longer - I believe you do not tend to go into a deep sleep when you know they are there. |
| Short naps/ Occasional | N= 4   1. Co-sleeping sometimes essential in first few days. Now baby is bigger will have occasional small nap beside him 2. Occasionally day sleep with bub or bring bub into my bed at 5am- too tired. Toddler I have is a terrible sleeper and I need to sleep when I can 3. avoiding co-sleeping - sometimes baby will only nap in contact with mum and mum gotta sleep sometime! 4. She has also slept in our bed on occasion (not regularly). It is difficult because you are tired and you do what works to get them to sleep! |
| Tradition (culture) | N= 3/292, 1%   1. convincing husband not to sleep with baby because everyone in his family does it 2. Co-sleeping and sleeping on their back- Being of Indonesian descent, most Indonesian parents co-sleep or put babies on their tummy to sleep. 3. not sleep with bub - I think natural to cuddle and sleep with baby. Husband African - they all sleep with baby there. Baby stops crying and breathes better when I snuggle him. |
| In the balance of risk, bedsharing seemed safe*r* | N= 3/292, 1%   1. It's sometimes difficult to resist the temptation to sleep with baby in my bed with me for at least short periods, especially when baby sleeps better that way and just a few more minutes sleep that it would result in seems so valuable. 2. not co-sleep, sometimes we all need sleep and it's more dangerous falling asleep while breastfeeding as you may drop the baby.   sleep in own bed - as a newborn the only way baby would settle was on top of me. Sitting up on a couch for hours wasn't a safe option so she came to bed |
| Health benefits for mum and bub | N= 2   1. no bedsharing - greatly under estimated for health benefits of bub and mother. most natural method on the planet. safe sleeping should be taught for bed sharing 2. not sleep with bub - I think natural to cuddle and sleep with baby. Husband African - they all sleep with baby there. Baby stops crying and breathes better when I snuggle him. |
| Bonding/ attachment/ (relationship for both parents) | N= 2/292,   1. Co-sleeping I believe is a form of bonding with your baby, my baby settles better in my bed 2. Avoiding co-sleeping - Its the only way to get rest sometimes in first few months and is good for bonding. |
| Closer monitoring when baby is sick/ post immunisations/ teething/ reflux | N= 2   1. Getting baby down to sleep in the bassinet. Due to baby already having had the flu, he slept in the bed with us in our king size bed in a controlled environment on a sleeping pad with walls for a long time and now is restless and upset in the bassinet.   often the baby wont settle when he's sick with a cold. I have had to sleep with him in my bed more often to help him settle and breathe. |
| *Environmental (no cot, no room) | N=1   1. No co-sleeping. Our room is not large enough to have a cot or bassinet and I prefer to have our baby sleep with us so I can monitor her closely at all times |
| Not wanting to wake other children/ other children waking baby | N= 1   1. No co-sleeping, baby was very clingy and constantly woken by older sibling's noise |
| Bedshared in hospital | N= 1   1. While stayed in Innisfail Hospital the staff were all very respectful and accommodating to our bedshare practice. Cairns hospital on the other hand were mostly rude and purposefully unaccommodating. |
|  | No reason provided- n= 64   1. Not to bedshare 2. It is hard to sleep baby separately 3. Co sleeping 4. CO-SLEEPING 5. I also co-sleep for some part of the night 6. Sharing a bed 7. Sleeping with bub 8. I co-sleep so baby is not in own bed 9. avoid sleeping in bed with you 10. I found it difficult to not bring baby in to our own bed to sleep in the middle of the night 11. Co-sleeping 12. Co sleeping with baby in bassinet in our room 13. during night, he sleeps next to me in my cot. he pulls over the blanket and covers his face and snuggles to breathe. so ensure blanket doesn't cover above his stomach 14. Co-sleeping sometimes 15. Not co-sleeping 16. don't co-sleep 17. also co-slept with baby 18. I co-sleep and share my doona with baby 19. co-sleeping - we co-slept with first child on and off and are doing so with this child when needed 20. sleeping in cot in the first month 21. Co-Sleeping 22. Particularly with first child co-sleeping became norm. 23. co-sleeping - me and my baby have always co-slept 24. sometimes we co-sleep with our baby 25. own bed - we co-sleep 26. Co-sleeping 27. We resorted to co-sleeping at times 28. Co-sleep at times 29. safe co-sleeping needs to be advertised and shown more 30. co-sleeping occasionally 31. same sleeping surface/co-sleeping 32. I know they don't recommend co-sleeping but I do sometimes 33. Sleeping only in crib 34. Avoid co sleeping. 35. Co sleeping 36. Baby co slept in our bed for the first 3 months 37. Co-sleeping 38. Co-sleeping, because I sleep with all three of my children in the same bed 39. co-sleeping with baby in adult bed 40. own blanket, no pillows - baby co-sleeps. I keep my pillow clear and well above baby. I often have our big blanket over her for extra warmth 41. because she ends up co-sleeping half way through the night 42. not co-sleeping 43. co-sleeping 44. co-sleep - we did this until 6 weeks then moved baby to her own room 45. he comes to bed (in our bed) with us at 6.30am 46. I co-slept with bub for first 4-6 weeks 47. co-sleeping helps all of us 48. own sleeping surface as often sleeps with me 49. we co-sleep for a few hours in the early morning 50. when baby is first born difficult to not sleep with baby on you 51. Only allowing them to sleep in crib, sometimes baby slept with us, on my chest or I made C shape around baby 52. to not share our bed with him while sleeping 53. we co-sleep for a few hours in the early morning 54. when baby is first born difficult to not sleep with baby on you 55. While stayed in Innisfail Hospital the staff were all very respectful and accomodating to our bedshare practice. Cairns hospital on the other hand were mostly rude and purposefully unaccommodating. 56. My baby sleeps beside me on my bed. It has been the best method for me and baby. 57. we co-sleep - baby in middle 58. I slept with the baby actually in my bed 59. So hard not to let him sleep in the bed with me. 60. sleeping in own cot 61. baby co-sleeps 62. Nil co-sleeping 63. separate sleep space, sleep on back, sleep at bottom of cot - baby had/has reflux so could not sleep on his back and still mostly sleeps in our bed 64. Co-sleeping in cold climate requires blankets (no insulation, sub-zero temps at night) |

Supplemental Table D: Full extraction table of free- text responses RQ1.3

| **Queensland Clinical Guidelines (QCG) + Risk Minimisation strategies (RM)** | **In line**  Free-text responses aligned with QCG guidance/ RM strategies |
| --- | --- |
| QCG: Increased risk: Excessive fatigue of caregiver  RM: ASSIST: Identify who parents could call on to help if extremely tired/unwell or planning to consume alcohol, medicine or drugs that may cause drowsiness • Include partners and other family members of household in conversations and planning where appropriate • Provide opportunity for verbal discussion to support safety strategies • Check understanding by asking carer to tell you in their words what the goals are • Recommend a follow up visit (with the same clinician if possible) within 2 weeks to review/revise the plan | 12/164= 0.07   1. Don't bed share if you're a heavy sleeper 2. Co-sleeping is not recommended (ie in the same bed), especially with both parents, if either parent is at risk of not waking up easily (eg due to alcohol or medication) and if the mattress is a pillowtop 3. bed sharing is not recommended for smokers, heavy sleepers, or if you have been drinking or any other substance 4. Ideally don't co-sleep but it you do there are lots of extra recommendations eg. make sure no one is overly tired or obese or under influence of drugs or alcohol 5. Don't co-sleep when tired 6. Don't co-sleep if heavy sleeper 7. do not share bed with heavy sleep/intoxicated or medicated adult 8. Not co-sleeping if tired/alcohol/drugs 9. Don't be a heavy sleeper when your co sleeping 10. no co-sleep if partner is in bed/ had a few drinks/ heavy sleeper 11. if co-sleeping - no siblings in bed, no drugs or alcohol, extreme fatigue 12. don't co-sleep when drunk/overtired/on medication |
| QCG: Increased risk: Sedative effect from caregiver intake of alcohol, medicine or drugs  RM: ASSIST: Identify who parents could call on to help if extremely tired/unwell or planning to consume alcohol, medicine or drugs that may cause drowsiness • Include partners and other family members of household in conversations and planning where appropriate • Provide opportunity for verbal discussion to support safety strategies • Check understanding by asking carer to tell you in their words what the goals are • Recommend a follow up visit (with the same clinician if possible) within 2 weeks to review/revise the plan | 82/164= 0.5   1. Not using drugs or alcohol while bed sharing 2. Don't co sleep (especially if anyone in the bed has been drinking alcohol, smoking, using any drugs) 3. No co-sleeping while taken medication or alcohol 4. Do not share a sleeping surface with baby if under the influence of drugs and alcohol. 5. if Co sleeping make sure drugs and alcohol aren't a factor 6. No cosleeping if taken drugs or alcohol 7. Co-sleeping is not recommended (ie in the same bed), especially with both parents, if either parent is at risk of not waking up easily (eg due to alcohol or medication) and if the mattress is a pillowtop 8. If co-sleep - dont share blankets, dont do after alcohol intake, if someone is smoker 9. bed sharing is not recommended for smokers, heavy sleepers, or if you have been drinking or any other substance 10. Ideally don't co-sleep but it you do there are lots of extra recommendations eg. make sure no one is overly tired or obese or under influence of drugs or alcohol 11. co-sleeping rules- not between parents, blankets covering face, parents not drinking alcohol and co sleep etc 12. Dont cosleep if drink/smoke etc - if do cosleep prctice safe cosleeping 13. Avoid having baby in co-sleeping arrangements with either mother or partner are smokers, have had alcohol or other drugs likely to make them drowsey 14. if co-sleeping, no drinking/drugs/smoking 15. Dont sleep with baby in bed when drinking or smoking 16. Do not share sleep surface if experiencing effects of drugs/medication that can cause drowsiness 17. don't bedsore if using pain killers/alcohol 18. co-sleep safely (dress warmly, advise other parent, no drugs/alcohol, no swaddle) 19. co-sleep - not drunk, obese -be careful 20. No sleeping with drunk parents 21. if co-sleeping do not be under the influence of any substances 22. if bed sharing- no alcohol or drugs 23. don't co-sleep while drunk 24. no drugs or alcohol if co-sleeping 25. no overweight, smoking, drinking adults sleeping with baby 26. no alcohol/drugs if co-sleeping 27. do not drink or do drugs and sleep with baby 28. no co-sleeping if smoker/alcohol 29. Don't co-sleep if drunk 30. No co-sleeping with alcohol onboard 31. No co-sleeping with intoxicated adult 32. don't sleep with baby under influence 33. do not share bed with heavy sleep/intoxicated or medicated adult 34. if in same bed no drugs, alcohol, bedding over head or sleeping bag 35. Not co-sleeping if tired/alcohol/drugs 36. don't sleep with baby if drinking, on drugs etc 37. parents not to sleep with baby especially if affected by alcohol 38. Avoid co-sleeping especially if drinker or smoker 39. Don't co-sleep intoxicated 40. don't consume substances and sleep with baby 41. if co-sleeping not under influence of meds etc 42. no drug or alcohol (parent and co sleeping) 43. do not sleep baby in bed with parents especially if any consumption of alcohol or drug 44. If co-sleeping don’t drink or smoke 45. No unsafe co-sleeping methods (alcohol/drugs) 46. if co-sleeping no smoking/drinking alcohol 47. not co-sleeping with parents using drugs/alcohol 48. don't sleep with family under influence of drugs/alcohol 49. Never cosleep with baby after alcohol 50. No smoking or alcohol especially if co sleeping 51. No co-sleeping in the middle of the bed between 2 adults - particularly if adults have been drinking alcohol 52. Sober parents if co-sleeping 53. Don't take baby to bed if you have consumed alcohol or prescription mess that cause drowsiness 54. Don't sleep near baby if been drinking/smoking 55. No bed sharing if had alcohol 56. Do not smoke or drink and sleep with or near baby 57. Don't sleep with baby if you have had alcohol or taken drugs 58. If co-sleeping, no smoking or alcohol 59. Do not bed share if smoke or drink alcohol 60. don't co-sleep under the influence of alcohol 61. do not have bub in bed if drinking 62. infant not sharing bed with smokers/drug users 63. no co-sleeping if smoking or/drinking 64. not co-sleeping when under influence 65. Don't co-sleep when drunk 66. Do not co-sleep with parents that smoke or drink 67. care giver not to sleep with bub if on alcohol or medication 68. no co-sleep if partner is in bed/ had a few drinks/ heavy sleeper 69. if co-sleeping - no siblings in bed, no drugs or alcohol, extreme fatigue 70. Dont bed share if under the influence 71. if bed sharing - no toddlers, intoxicated or drowsy medication for adults 72. if co-sleeping avoid drugs/alcohol 73. don't co-sleep if you drink/smoke 74. Follow co-sleeping rules ie. no alcohol/no duvet near baby 75. do not co-sleep if affected by alcohol/drugs 76. if co-sleeping - only with no alcohol or drugs in system 77. don't co-sleep when drunk/overtired/on medication 78. no co-sleeping on soft surfaces, couches etc with adult who smokes, drinks, medication etc 79. no co-sleeping if been drinking, smoking with children etc 80. Don’t drink and co-sleep 81. no co-sleeping if under the influence 82. Dont co-sleep if drinking alcohol |
| QCG: Increased risk: Antenatal and postnatal exposure to tobacco smoke  RM: ASSIST: • Support conversations with caregivers to further identify safe sleeping goals • Conversation prompts may include -How to keep the room that infant sleeps in smoke-free | 32/164= 0.195   1. Don't let the baby sleep in the bed if one of the parents smokes 2. If co-sleep - dont share blankets, dont do after alcohol intake, if someone is smoker 3. Don't co sleep (especially if anyone in the bed has been drinking alcohol, smoking, using any drugs) 4. Do not sleep with your child if you are a smoker. 5. bed sharing is not recommended for smokers, heavy sleepers, or if you have been drinking or any other substance 6. Dont cosleep if drink/smoke etc - if do cosleep prctice safe cosleeping 7. Avoid having baby in co-sleeping arrangements with either mother or partner are smokers, have had alcohol or other drugs likely to make them drowsey 8. if co-sleeping, no drinking/drugs/smoking 9. dont co sleep with parent who smokes 10. Dont sleep with baby in bed when drinking or smoking 11. don't share bed with parent who smokes 12. No sleeping with smokers 13. Don't co-sleep if a smoker 14. no overweight, smoking, drinking adults sleeping with baby 15. no co-sleeping if smoker/alcohol 16. No co-sleeping with smoker 17. Avoid co-sleeping especially if drinker or smoker 18. no co-sleeping if mum or dad are smokers 19. If co-sleeping don’t drink or smoke 20. if co-sleeping no smoking/drinking alcohol 21. not to co-sleep with smoking parents 22. No smoking or alcohol especially if co sleeping 23. Don't sleep near baby if been drinking/smoking 24. Do not smoke or drink and sleep with or near baby 25. If co-sleeping, no smoking or alcohol 26. Do not bed share if smoke or drink alcohol 27. infant not sharing bed with smokers/drug users 28. no co-sleeping if smoking or/drinking 29. Do not co-sleep with parents that smoke or drink 30. don't co-sleep if you drink/smoke 31. no co-sleeping on soft surfaces, couches etc with adult who smokes, drinks, medication etc 32. no co-sleeping if been drinking, smoking with children etc |
| QCG: RM: Consider the size of the sleep surface in relation to the people sharing the space–is there enough room to create a safe clear space for infant sleep | 9/164= 0.05   1. Safe co sleeping with a side co sleeper 2. If co-sleeping use a co-sleeper 3. using a bassinet/ co sleeper 4. sleeping in co-sleeper 5. if in bed with baby use a (bed) baby sleeper to protect them 6. Use a co-sleeper bed when sleeping baby next to adults 7. Co sleep bassinet 8. Baby to sleep in own bassinet/cot or if bed sharing in a separate little bed 9. SIDS approved side sleeper |
| QCG: Increased risk: Environments with entrapment hazards (e.g. sofas/couches and armchairs)  RM: Falling asleep holding infant on a couch/chair is a major risk for infant suffocation through entrapment - If considered a possibility, recommend caregivers move themselves and infant to a safer sleep environment (e.g. firm flat level mattress) | 17/164= 0.103   1. Don't co sleep or nap with baby on bed or couch 2. Don't sleep on couches, sofas etc 3. Dont sleep on chairs/sofas 4. careful not to fall asleep on couch 5. No sleeping on couches (or soft surfaces that could smother baby) 6. don't sleep on couch/holding them 7. Don't sleep on couches with bub 8. not holding sleeping baby on chair, lounge 9. not asleep on couch/chair w/mum 10. Don't fall asleep on couch 11. Never sleep baby on couch/beanbag/soft surfaces 12. Don't sleep in chair or on sofa with baby 13. don't sleep them on a couch 14. Do not sleep in chair/sofa 15. no co-sleeping on couch 16. no co-sleeping on soft surfaces, couches etc with adult who smokes, drinks, medication etc 17. No sleeping on couch with bub |
| QCG: Increased risk: Soft sleep surfaces (e.g. pillows, doonas)  RM: o Mattress is firm, flat and level -Keep pillows and bedding positioned away from infant sleep space -Avoid doonas and duvets as increases risk of accidental head covering -Ideally provide infant with separate infant bedding or use an infant sleeping bag to avoid use of adult bedding -Avoid use of any soft sleep surface for example bean bag, waterbed, sagging mattress, soft in-bed sleep devices or positioners | 4/164= 0.02   1. Co-sleeping is not recommended (ie in the same bed), especially with both parents, if either parent is at risk of not waking up easily (eg due to alcohol or medication) and if the mattress is a pillowtop 2. No sleeping on couches (or soft surfaces that could smother baby) 3. Never sleep baby on couch/beanbag/soft surfaces 4. no co-sleeping on soft surfaces, couches etc with adult who smokes, drinks, medication etc |
|  | QCG: Increased risk: Infant movement is restricted (e.g. infant wrapped/swaddled)  RM: Infant is not wrapped as this restricts arm and leg movement • If used, remove necklaces, hooded clothing before settling to sleep • Ideally, use an infant sleep suit or sleeping bag to keep infant warm and reduce need for additional bedding or use of adult bedding  8/164= 0.048   1. Not swaddling while in bed with parents 2. Cosleep unswaddled 3. Don't swaddle if bed sharing 4. No co-sleeping -Don’t wrap baby if co-sleeping 5. co-sleep safely (dress warmly, advise other parent, no drugs/alcohol, no swaddle) 6. no wrapping of the baby when sleeping next to adults 7. if co-sleeping - do not swaddle or share blankets   do not swaddle if co-sleepign |
| QCG: Increased risk: Soft sleep surfaces (e.g. pillows, doonas)  RM: Keep pillows and bedding positioned away from infant sleep space o Avoid doonas and duvets as increases risk of accidental head covering o Ideally provide infant with separate infant bedding or use an infant sleeping bag to avoid use of adult bedding | 30/164= 0.182   1. if bed sharing, baby to have own blanket 2. If co-sleep - dont share blankets, dont do after alcohol intake, if someone is smoker 3. co-sleeping rules- not between parents, blankets covering face, parents not drinking alcohol and co sleep etc 4. Don't put baby under parents doona 5. If co sleeping don't share blanket with baby 6. co-sleep safely (dress warmly, advise other parent, no drugs/alcohol, no swaddle) 7. No loose bed linen near baby 8. if co-sleeping own blankets and not in middle of 2 parents 9. No blankets/pillows in bed 10. if co-sleeping/bed sharing - baby uses own blankets 11. if co-sleeping use own blanket 12. if co-sleeping, no blankets 13. if in same bed no drugs, alcohol, bedding over head or sleeping bag 14. if co-sleeping ensure baby has their own blanket 15. if co-sleeping no loose pillows/bedding 16. if in bed with you lay on back - don't share a blanket 17. if co-sleeping no loose blankets 18. Keep baby seperate from bed doona or blanket (If co-sleeping) 19. Avoid blanket sharing 20. baby has own blankets 21. don't put anything (comforter) where they can pull over face 22. if co-sleeping - do not swaddle or share blankets 23. if co-sleeping baby has its own blanket 24. baby has his own bedclothes 25. if cosleeping avoid blankest and pillow 26. Follow co-sleeping rules ie. no alcohol/no duvet near baby 27. if co-sleeping no adult blankets near baby 28. If co-sleeping no under mums blankets 29. keeping pillows away from baby 30. No pillows |
| QCG: Increased risk: Person with obesity sharing the sleep surface  RM: Consider the size of the sleep surface in relation to the people sharing the space–is there enough room to create a safe clear space for infant sleep | 4/164 = 0.02   1. Ideally don't co-sleep but it you do there are lots of extra recommendations eg. make sure no one is overly tired or obese or under influence of drugs or alcohol 2. co-sleep - not drunk, obese -be careful 3. no overweight, smoking, drinking adults sleeping with baby 4. Don't sleep with baby if you are severly overweight |
| QCG: Increased risk: Multiple bed-sharers, including siblings and pets  RM: Place infant to the side of one parent -Avoid placing infant between parents | 16/164= 0.097   1. co-sleeping rules- not between parents, blankets covering face, parents not drinking alcohol and co sleep etc 2. Co-sleeping is not recommended (ie in the same bed), especially with both parents, if either parent is at risk of not waking up easily (eg due to alcohol or medication) 3. if co-sleeping own blankets and not in middle of 2 parents 4. never sleep between two adults 5. bedsharing - sleep baby on outside of bed 6. if co-sleeping/bed sharing - baby sleeps on outside of bed not in-between parents 7. do not sleep infant between adults 8. If co-sleeping, baby on outside 9. if co-sleeping, baby not to be between parents 10. Baby to sleep next to mum only (Not between mum and dad) 11. No co-sleeping in the middle of the bed between 2 adults - particularly if adults have been drinking alcohol 12. Don’t sleep with baby between parents in the bed 13. if cosleeping not between you and partner 14. Sleep the baby beside one parent only 15. No sleep in between mum and dad 16. don't put baby between 2 people |
| QCG: Increased risk: Multiple bed-sharers, including siblings and pets  RM: Place infant to the side of one parent -Avoid placing infant between parents -Avoid placing infant next to other children or pets | 11/164= 0.067   1. Do not co sleep with baby if other children are in the bed 2. No siblings in bed 3. don't bedsore with other kids 4. Do not let baby sleep on an adult sleeping surface with other children 5. kids are not to share the bed with the baby 6. if co-sleeping - no siblings in bed, no drugs or alcohol, extreme fatigue 7. if bed sharing - no toddlers, intoxicated or drowsy medication for adults 8. If co-sleeping, no pets or other children 9. Never place the baby to sleep with other children 10. away from toddlers 11. no co-sleeping if been drinking, smoking with children etc |
| QCG: Increased risk: Multiple bed-sharers, including siblings and pets  RM: Place infant to the side of one parent -Avoid placing infant between parents - Avoid placing infant next to other children or pets | 3/164= 0.018   1. No pets in bed if sharing 2. If co-sleeping, no pets or other children   no pets present |
| QCG- not currently included but would sharpen guidance | 1. co-sleep safely (dress warmly, advise other parent, no drugs/alcohol, no swaddle) |
| QCG: Increased risk: If the caregiver falls asleep with the infant in this position, the risk of SUDI from entrapment and/or suffocation against the carers body or against soft bedding is significantly increased  RM: Place infant on their back for every sleep | 3/164= 0.018   1. don't sleep on couch/holding them 2. don't fall asleep with baby on you 3. don't sleep with a baby on you |
| QCG: Increased risk: Environments with entrapment hazards (e.g. sofas/couches and armchairs  RM: -Mattress is firm, flat and level | 1/164= 0.006   1. if co-sleeping, safe flat surface |
| QCG: Increased risk: Prone sleep position of infant  RM: Place infant on their back for every sleep | 1/164= 0.006   1. if in bed with you lay on back - don't share a blanket |
| QCG: Increased risk: Greatest risk between 2–4 months | 1/164= 0.006   1. Co-sleeping after 3 months (bed sharing) |
| **Queensland Clinical Guidelines (QCG) + Risk Minimisation strategies (RM)** | **Not in line**  Free-text responses that do not align with QCG guidance/ RM strategies |
| QCG: Increased risk: Soft sleep surfaces (e.g. pillows, doonas) | 1/164= 0.006   1. If co-sleeping have baby in co-sleep nest |
| QCG: Increased risk: Prone sleep position of infant  RM: Place infant on their back for every sleep | 1/164= 0.006   1. Don’t have baby lying on stomach until neck is strong enough |
| QCG: Increased risk: Environments with entrapment hazards  RM: • To minimise risk of fall or entrapment, recommend -Place infant away from edge of bed or place mattress on the floor -Move bed or mattress away from wall so infant cannot become trapped/wedged between the bed and the wall | 1/164= 0.006   1. Bedrail if co-sleeping |
| **Queensland Clinical Guidelines (QCG) + Risk Minimisation strategies (RM)** | **Ambiguous**  Free-text responses that could not be identified as aligned nor not aligned due to ambiguous nature of response |
|  | 1. don't sleep on dangerous surfaces   Do not feed on couch/sofa in case of falling asleep |
|  | 1. don't co-sleep with partner 2. No co-sleeping with both parents 3. no co-sleep if partner is in bed/ had a few drinks/ heavy sleeper |
| QCG: Increased risk: Environments with entrapment hazards  RM: • To minimise risk of fall or entrapment, recommend -Place infant away from edge of bed or place mattress on the floor -Move bed or mattress away from wall so infant cannot become trapped/wedged between the bed and the wall | 5/164= 0.03   1. Mother position if co-sleeping 2. position yourself so you can't roll over 3. if co-sleeping, move away from baby 4. if co-sleeping - baby should be away from edge and middle 5. If co-sleeping have baby on side facing out |
|  | 1. Don't let baby fall asleep on parents' chest 2. Not sleeping on parents 3. put the child on your chest until they sleep then take them to the bed |
|  | 37/164= 0.225   1. Follows SIDS recommendations if cosleeping 2. Practice safe co sleeping if co sleeping 3. co-sleeping rules- not between parents, blankets covering face, parents not drinking alcohol and co sleep etc 4. Dont cosleep if drink/smoke etc - if do cosleep prctice safe cosleeping 5. use safe co-sleeping methods 6. Follow recommendations for safe cosleeping 7. Practice safe co sleeping (if applicable) 8. If co-sleeping adopt safe co-sleeping practices 9. dos and donts of cosleeping 10. co-sleep safely (dress warmly, advise other parent, no drugs/alcohol, no swaddle) 11. co-sleep - not drunk, obese -be careful 12. bed share (as per safe sleeping) 13. co-sleeping safety rules 14. if co-sleeping, follow safe sleeping guidelines 15. if co-sleeping, safe flat surface 16. Safe bed sharing as baby regulates breath to mother 17. safe bed sharing 18. We have safely co-slept from day 1 19. co-sleep safely 20. follow co-sleeping guidelines if putting in your bed 21. Co sleeping can increase risk, but there are safer ways of doing it 22. If you are going to co sleep, do it safely 23. Precautions for co-sleeping 24. Safe cosleeping 25. Co sleeping safety 26. Baby to sleep in parents rom for first 6 months in cot or safe bedsharing option 27. co-sleep safely and use safe guidelines 28. Safe co-sleeping 29. follow safe co-sleeping advice 30. If co-sleeping do so safely 31. Follow co-sleeping rules ie. no alcohol/no duvet near baby 32. Follow safe bedsharing guidelines, safe sleeping guidelines 33. cosleep with conditions 34. Practice co-sleeping safely 35. safe co-sleeping 36. Avoid co-sleeping but if you do follow the guidelines 37. Co-sleeping sleeping with baby |
